# MYC—Master Regulator of the Cancer Epigenome and Transcriptome

**DOI:** 10.3390/genes8050142

**Published:** 2017-05-13

**Authors:** Candace J. Poole, Jan van Riggelen

**Affiliations:** Augusta University, Department of Biochemistry and Molecular Biology, 1410 Laney-Walker Blvd., Augusta, GA 30912, USA; capoole@augusta.edu

**Keywords:** MYC, chromatin remodeling, cancer

## Abstract

Overexpression of *MYC* is a hallmark of many human cancers. The *MYC* oncogene has long been thought to execute its neoplastic functions by acting as a classic transcription factor, deregulating the expression of a large number of specific target genes. However, MYC’s influence on many of these target genes is rather modest and there is little overlap between MYC regulated genes in different cell types, leaving many mechanistic questions unanswered. Recent advances in the field challenge the dogma further, revealing a role for MYC that extends beyond the traditional concept of a sequence-specific transcription factor. In this article, we review MYC’s function as a regulator of the cancer epigenome and transcriptome. We outline our current understanding of how MYC regulates chromatin structure in both a site-specific and genome-wide fashion, and highlight the implications for therapeutic strategies for cancers with high MYC expression.

## 1. Introduction

The importance of MYC in human development and disease has generated intense research interest over the past 30 years, resulting in numerous original articles and reviews (for example, see [1,2,3]). The *MYC* family is comprised of *c-MYC* (herein referred to as *MYC*, unless otherwise specified), *N-MYC*, and *L-MYC*, which encode for basic helix–loop–helix leucine zipper (bHLH-Zip) transcription factors [4] that have been found to play a unique role in regulating an extensive range of biological processes including stemness, cellular proliferation, and neoplastic transformation. While the activity of MYC family members is tightly regulated in non-malignant cells, their constitutive expression is directly linked to the pathogenesis of a wide variety of human cancers [5,6,7,8]. The fact that elevated levels of MYC proteins are found in 60–70% of all cancers (reviewed in [1]) and the discovery that tumors can be dependent on continuous MYC expression (known as oncogene addiction) [9] have made this family of oncogenes a highly promising therapeutic target. However, even after three decades of research, both the exact molecular mechanism of how MYC promotes tumorigenesis and a pharmacologic inhibitor selectively targeting the oncogene remain elusive.

**Figure 1:**
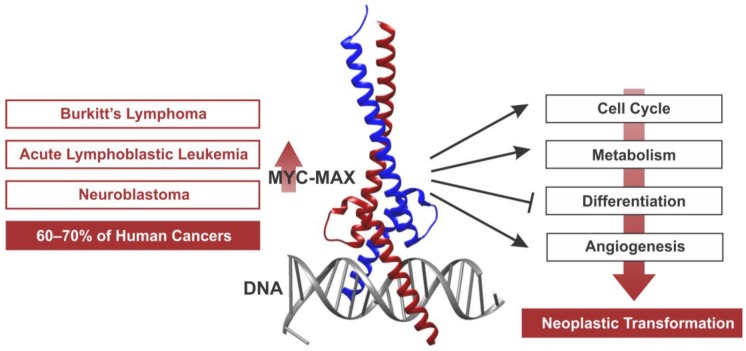
MYC as a transcription factor and oncogene. Display of the X-ray crystal structure of a MYC–MAX heterodimer bound to DNA as a site-specific transcription factor complex [20,21]. Overexpression of MYC causes the deregulation of central cellular processes including cell cycle progression, metabolism, differentiation, and angiogenesis, together contributing to neoplastic transformation. Deregulated MYC expression is implicated in a wide variety of human cancer types including Burkitt’s lymphoma, acute lymphoblastic leukemia (ALL), and neuroblastoma. Image created with: UCSF Chimera; PDB: 1NKP.

MYC exerts its neoplastic features by increasing autonomous cellular proliferation, growth, angiogenesis, and genomic destabilization while blocking differentiation (see Figure 1) (reviewed in [10]). However, these diverse cellular functions are attributed to the still not completely understood ability of MYC to control the expression of a large set of genes. MYC proteins have first been described as sequence-specific transcription factors, forming heterodimeric complexes with MYC-Associated Factor X (MAX). MYC–MAX complexes are now known to recognize a consensus sequence known as Enhancer box (“E-box”), activating the transcription of genes [11,12,13]. This finding sparked a comprehensive search for MYC target genes and their function and involvement in neoplastic transformation [14]. However, the scope and complexity of MYC’s action became apparent when it was discovered that there are approximately 20,000 E-box sites in the human genome, of which only a subset is differentially bound by MYC in a cell type-specific fashion [15]. To further increase the complexity, MYC was also later found to be capable of repressing the transcription of genes through interactions with other transcription factors [16,17,18]. It turned out that both MYC’s activating and repressing functions are critical for tumorigenesis and depend on the recruitment of chromatin modifying co-factors that remodel chromatin structure in the vicinity of the binding sites. However, MYC acts rather weakly at many of its target gene promoters, and even genomic location profiles have often proven non-predictive of MYC-dependent transcriptional regulation [15]. Furthermore, despite considerable efforts to identify a MYC target gene signature using comparative gene profiling and genomic location analyses, little overlap between MYC regulated genes in different cell types has been found [14,19]. To explain these discrepancies, the classic mechanistic model has recently been extended to incorporate MYC’s function as a regulator of global chromatin structure and transcription. In this article, we review the role of MYC as a regulator of the cancer epigenome and transcriptome both through site-specific, local mechanisms as well as genome-wide effects, and highlight the potential for novel therapeutic strategies.

## 2. Recruitment of Chromatin Modifiers for MYC-Dependent Transactivation

The gene-specific transactivation model depends on the ability of MYC–MAX complexes to recruit chromatin-modifying co-factors. These change the chromatin structure in the vicinity of the binding site, allowing or preventing the transcription of the corresponding gene by regulating its accessibility to the basal transcriptional machinery or releasing preloaded RNA Polymerase II (RNA Pol II) from pausing.

**Figure 2:**
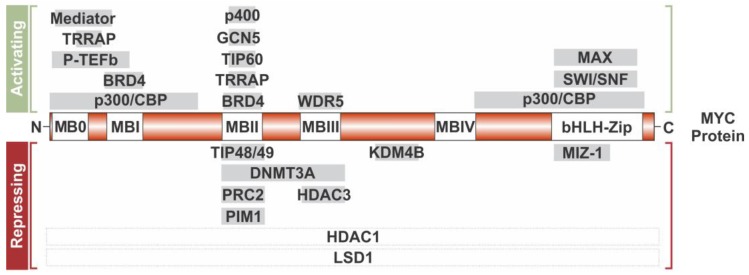
Chromatin modifying co-factors interacting with the MYC oncoprotein. MYC’s N-terminus harbors the transcriptional activation domain (TAD) domain and MYC box (MB) 0, I, and II; MB III and IV are in the central MYC domain; and MYC’s C-terminus holds the basic helix–loop–helix leucine zipper (bHLH-Zip) domain. These regions facilitate protein–protein interactions with multiple chromatin modifiers for activation and repression. The N-terminus associates with co-activators: Mediator, Transactivation/Transformation-Associated Protein (TRRAP), Positive Transcription Elongation Factor b (P-TEFb), Bromodomain-Containing Protein 4 (BRD4), and E1A Binding Protein p300/CREB Binding Protein (p300/CBP) ; while MB II associates with coactivators: E1A-Binding Protein p400 (p400), General Control of Amino Acid Synthesis Protein 5-Like 2 (GCN5), 60kDa Tat Interacting Protein (TIP60), TRRAP, BRD4; as well as co-repressors: 48 KDa TBP-Interacting Protein (TIP48)/49 KDa TBP-Interacting Protein (TIP49), Polycomb Repressive Complex 2 (PRC2), and Pim-1 Oncogene/Pro-viral Integration Site 1 (PIM1). The co-repressor, DNA Methyltransferase 3a (DNMT3A), associates with MB II and MB III while Histone Deacetylase 3 (HDAC3) associates only with the MB III region. WD Repeat Domain 5 (WDR5) interacts with MB III. The co-activator, Lysine (K)-Specific Demethylase 4 (KDM4B), associates with MYC’s central region. Coactivators: MYC Associated Factor X (MAX), Interactor 1 Protein (INI1) (part of the SWI/SNF complex), and p300/CBP associate with the bHLHZip domain, as well as the co-repressor, Myc-Interacting Zinc Finger Protein 1 (MIZ-1). Dotted lined boxes (Histone Deacetylase 1 (HDAC1) and Lysine-Specific Histone Demethylase 1A (LSD1) indicate association with MYC, even though the exact interaction domain is unknown.

An increasing number of chromatin modifying co-factors including chromatin “writers”, “readers”, and “erasers” have been found to interact directly or indirectly with MYC–MAX complexes (for an overview, see Figure 2). Many of these protein–protein interactions are facilitated through MYC’s N-terminus, which harbors the transcriptional activation domain (TAD) and highly conserved sequence elements, known as “MYC box” (MB) 0, I, and II, followed by MB III and IV in the central MYC domain ([22] and reviewed in [23]). MB I, II, and III are essential for all biological functions of MYC, and are required for site-specific transactivation and transrepression of most, but not all, direct target genes [24,25]. The exact mechanism of how MYC coordinates all these protein–protein interactions and how individual chromatin modifying enzymes contribute to MYC’s oncogenic properties is subject to intense research, but still not completely understood. While the recruitment of activating vs. repressing factors is thought to be determined by additional DNA-binding proteins such as Specificity Protein-1 (SP1) or MYC-Interacting Zinc Finger Protein-1 (MIZ-1), it is not clear whether variants of the E-box motif found in different sets of target genes influence the quality of protein–protein interactions. Recent research has been focused on local chromatin structure, which facilitates tethered recruitment, as discussed below, but might also play a role in defining the type of co-factor interaction. Besides the possibility that specific co-factors are recruited in a context-dependent manner, they also might be recruited in response to stimulation, allowing for an additional level of signal integration to modulate the transcriptional output of the MYC network.

### 2.1. Histone Acetyltransferases and MYC-Dependent Transactivation

**Figure 3:**
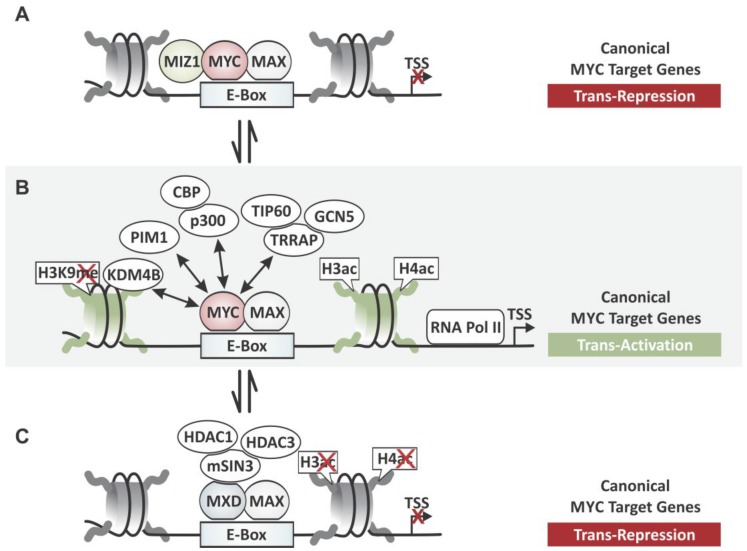
Site-specific transcriptional activation of target genes through the MYC network. (**A**) The ratio of MYC–MIZ-1 and MYC–MAX at the enhancer box (E-box) determines whether a gene is activated or repressed. MIZ-1 can be recruited to form a trimeric complex with MYC–MAX that represses genes as an additional level of regulation; (**B**) MYC forms heterodimeric complexes with MAX binding E-box sequences to transactivate canonical target genes through recruitment of chromatin modifying co-factors. The KDM4 demethylase removes the inactive histone 3 lysine 9 tri-methylation (H3K9me3) mark for transactivation. PIM1 kinase phosphorylates nucleosomes at histone 3 serine 10 (H3S10ph) locally for activation and phosphorylates MYC itself to enhance protein stability. TIP60 and GCN5 via TRRAP and p300/CBP act as histone acetyltransferases (HATs) increasing acetylation of histone H3 and H4 (H3ac and H4ac) in the vicinity of the binding site, inducing an open chromatin conformation, allowing RNA Polymerase II (RNA Pol II) machinery to bind the core promoter. (**C**) MYC’s transactivating function is antagonized by MXD–MAX heterodimers that compete with MYC–MAX for E-box binding, but repress canonical target genes through recruitment of HDACs (HDAC1 and HDAC3) via scaffolding protein SIN3 Transcription Regulator Family Member A (mSIN3), resulting in local deacetylation of histone H3 and H4. TSS: Transcription start site.

The site-specific transactivation of canonical target genes containing one or more E-box sequences is the best characterized mechanism of how MYC–MAX complexes control transcription (see Figure 3B). When bound to E-box sites, MYC is known to recruit the adaptor protein, Transactivation/Transformation-Associated Protein (TRRAP) [26,27]. TRRAP is a component of many histone acetyltransferase (HAT) complexes including SPT3-TAF(II)31-GCN5L acetylase coactivator complex (STAGA) (SPT-ADA-GCN5 acetylase coactivator complex (SAGA) in yeast), and contributes to the transformation activity of MYC through interactions with MB I and II [26,27]. It serves as a scaffold for assembling multiprotein complexes, recruiting the HATs, General Control of Amino Acid Synthesis Protein 5-Like 2 (GCN5) [26], and 60kDa Tat Interacting Protein (TIP60) [28], as well as the Switch/Sucrose Non-Fermentable (SWI/SNF)-related histone-exchange protein, E1A-Binding Protein p400 (p400) [29], which interacts with MYC indirectly. The finding that TRRAP is necessary for malignant transformation by MYC reveals the importance of this transactivation mechanism [26,27]. Upon recruitment, GCN5 preferentially acetylates lysines 9, 14, and 18 of histone H3 (H3K9/14/18), whereas TIP60 acetylates lysines 5, 8, and 12 of histone H4 (H4K5/8/12) and lysine 5 of histone H2A (H2AK5) thereby loosening the interaction between nucleosomes and DNA in the vicinity of the binding site, allowing RNA Pol II to access the core promoter, ultimately leading to transcription of the corresponding genes [30,31,32]. 

In parallel or as an alternative mechanism, MYC recruits E1A Binding Protein p300/CREB Binding Protein (p300/CBP) as a stabilizing co-activator that promotes transactivation of canonical target genes [33]. p300 and CBP activate transcription by opening chromatin structure through their intrinsic HAT activity, by acting as adaptor molecules for additional co-factors, and by recruiting the basal transcriptional machinery [34,35]. MYC directly recruits p300/CBP via its TAD, independent of the adaptor protein, TRRAP [28,33]. However, p300/CBP has also been shown to interact with MYC’s C-terminus, suggesting additional as yet unknown functions of p300/CBP at distinct promoters [36]. Moreover, p300 has a dual role in regulating MYC’s activity. In addition to its function as a co-activator of MYC-dependent transcription, p300 regulates MYC protein turnover [33]. p300 acetylates MYC at several lysine residues located between the TAD and DNA-binding domain at the C-terminus. While p300/CBP binding has been demonstrated to stabilize MYC protein independently of acetylation, p300-mediated acetylation of MYC results in its increased proteasomal degradation [33,37]. Conversely, MYC protein stability can be increased through expression of GCN5 or TIP60, indicating distinct roles of HATs in regulating MYC’s functions, even though in the latter case it is not known if this requires HAT activity [38]. This indicates that MYC not only serves as a hub for co-factors, but that MYC itself is regulated by these enzymes. Whether any of the acetylation sites facilitate the binding of specific interaction partners to MYC remains to be seen. While the recruitment of GCN5/TIP60/p300/CBP is associated with a variety of acetylation marks [19,39], various combinations of these activating marks have been observed at different loci, suggesting that MYC might recruit distinct co-factors to certain promoters. Their levels generally correlate with transcriptional activity, which is consistent with the recent finding that MYC enhances the expression of already active genes to boost the transcriptome of a given cell [40,41]. 

### 2.2. Histone Demethylases and MYC-Dependent Transactivation

In addition to histone acetyltransferases, there is increasing evidence that MYC recruits lysine-specific histone demethylases (KDM) to activate transcription of canonical target genes, even though the exact mechanism of how histone methylation contributes to MYC regulated gene expression remains unknown. First hints that MYC-dependent transactivation involves KDMs came from *Drosophila* MYC (dMYC). The Trithorax group protein dKDM5/LID that belongs to the JARID1 family of histone H3 lysine 4 (H3K4) demethylases was found crucial for dMYC-promoted cell growth [42]. However, since H3K4 methylation is an active chromatin mark, it seemed counter intuitive that dKDM5/LID is recruited for transactivation. The subsequent finding that dMYC actually negatively regulates dKDM5/LID activity, shed some light on this matter and led to the speculation that dKDM5/LID may facilitate dMYC binding to chromatin or play a role in preserving H3K4 methylation marks, although this needs further study. More recently, MYC has been reported to directly interact with Lysine (K)-Specific Demethylase 4 (KDM4B) and recruit the histone demethylase to E-box target genes (see Figure 3B) [43,44]. KDM4B interacts with the central region of N-MYC (amino acids 99–300) [44]. It specifically demethylates lysine 9 of histone H3 (H3K9me3/me2), removing repressive chromatin marks, thereby contributing to gene activation [45]. This mechanism was reported for MYC in embryonic stem cells (ESCs) and for overexpressed N-MYC in neuroblastoma [43,44], indicating that the decreased H3K9me3 deposition plays a role for both MYC’s physiologic as well as its oncogenic function. While the elevated expression of KDM4B in N-MYC amplified neuroblastomas is associated with poor clinical outcome, inhibition of KDM4B suppresses MYC function. Loss of KDM4B function causes downregulation of N-MYC target genes, subsequently inhibits cellular proliferation, induces differentiation, and delays neuroblastoma tumor growth. This indicates that MYC alters histone methylation patterns in the vicinity of E-box sites, preserving or even accumulating active marks such as H3K4 methylation, while decreasing inactive marks such as H3K9 methylation.

### 2.3. Protein Kinases and MYC-Dependent Transactivation

Another chromatin modifying co-factor that MYC recruits to E-box target genes is the Proviral Integration Site 1 (*PIM1*) oncogene, a constitutive active serine/threonine kinase (see Figure 3B). After stimulation with growth factors, PIM1 forms a complex with MYC–MAX via MB II [46]. Subsequent PIM1-dependent phosphorylation of histone H3 on serine 10 (H3S10ph) in the vicinity of E-box sites has been shown to contribute to the activation of approximately 20% of the MYC-regulated genes and neoplastic transformation of Rat-1 fibroblasts [46]. H3S10ph is thought to stimulate RNA Pol II recruitment and release from promoter-proximal pausing [47]. Furthermore, PIM1 can phosphorylate MYC at serine 62 while decreasing threonine 58 phosphorylation, thereby increasing MYC protein half-life [48]. Both residues are known as a main switch controlling MYC protein stability and proteasomal degradation [49,50]. Similarly, Proviral Integration Site 2 (PIM2) synergizes with MYC, stabilizing MYC protein through phosphorylation of serine 329 [48]. PIM kinases were first identified as genes that cooperate with *Eµ-myc* in lymphomagenesis, an observation that later could be extended to various cancer types including pre-B-cell lymphoma, prostate carcinomas and triple-negative breast cancer [51,52,53]. Together, this indicates that PIM kinases cooperate with MYC during tumorigenesis by increasing MYC’s transcriptional activity for some target genes through multiple mechanisms, including modifying the phosphorylation status of MYC to enhance its activity and stability, as well as activating local chromatin structure in the vicinity of MYC binding sites in a signal-dependent fashion. Hence, PIM kinases have sparked interest as a molecular target in multiple cancer types including lymphomas and prostate cancer.

### 2.4. The Role of ATP-Dependent Chromatin Remodeling in MYC-Dependent Transactivation

An early connection between MYC and chromatin structure is the interaction with Integrase Interactor 1 Protein (INI1), a core subunit of the SWI/SNF chromatin remodeling complex [54,55]. The SWI/SNF complex mobilizes nucleosomes in an ATP-dependent fashion by catalyzing the exchange of histone octamers allowing for DNA to become accessible to transcriptional machinery (reviewed in [56]). The interaction with the SWI/SNF complex has been shown to be important for MYC-dependent transcription and transformation [54,55]. MYC’s bHLHZip domain directly interacts with INI1 and recruits the SWI/SNF complex to E-boxes for transactivation [54,57]. This interaction was found independent of MYC–MAX binding despite both binding to MYC’s bHLHZip domain, indicating both activating mechanism occur in parallel. INI1 is a tumor suppressor that interacts with many other proteins, including oncogenes and tumor suppressor genes. INI1 is frequently mutated in a wide variety of cancers and its loss is associated with neoplastic transformation [58]. Interestingly, INI1 and MYC act antagonistically on a subset of target genes including genes involved in cell cycle progression, metabolism, and ribosomal biogenesis, suggesting that INI1 negatively regulates MYC binding and/or transcriptional activity. Highlighting the importance of this mechanism, re-expression of INI1 negatively affected proliferation of MYC-positive INI1-deficient rhabdoid tumor cells [55]. Additional investigations are needed to identify MYC- and SWI/SNF-dependent target genes and to unravel their molecular mechanisms, specifically how they work together to contribute to neoplastic transformation.

### 2.5. Models for Antagonizing MYC-Dependent Transactivation

The transactivation of E-box target genes by MYC–MAX can be antagonized by MAX-Dimerization (MXD) proteins. MXD family members such as MXD1 and MAX Network Transcriptional Repressor (MNT) also form heterodimeric complexes with MAX, competing with MYC–MAX for binding to the same E-box sequences, but subsequently repress the corresponding gene [59,60]. While under non-malignant conditions an equilibrium exists that is defined by the relative abundance of MYC and MXD proteins, the constitutively elevated expression of MYC shifts the balance toward activation in tumor cells. The MXD-dependent repression mechanism relies on the recruitment histone deacetylases (HDACs), such as HDAC1 and HDAC3, which reduce histone acetylation on local chromatin resulting in a more condensed nucleosomal conformation, through the adapter protein SIN3 Transcription Regulator Family Member A (mSIN3) (see Figure 3C) [61]. The recruitment of co-repressors is essential for all the cellular functions of MXD proteins. The recruitment of HATs by MYC/MAX complexes and HDACs by MXD/MAX complexes can be seen as a transcriptional switch that regulates the activity of canonical MYC target genes through histone acetylation, shifting an equilibrium by “opening” or “closing” the local chromatin structure, allowing or preventing RNA Pol II binding, respectively. 

Adding another level of complexity, the transactivating effect of MYC–MAX on E-box regulated genes has recently been found to be antagonized by MIZ-1 [62]. While the interaction between MYC and MIZ-1 has been known to transrepress non-canonical MYC target genes that contain the initiator element (INR) sequence motif (see Chapter 3), recent genome-wide location profiling revealed that MIZ-1 also interacts with MYC–MAX complexes occupying E-box sites [62]. The relative amounts of MYC and MIZ-1 that are bound to the core promoter determine whether the gene is activated or repressed [62]. While MYC-repressed E-box genes were characterized by a MYC/MIZ-1 ratio close to 1, MYC-activated genes showed higher ratios. Interestingly, in tumor cells with high MYC expression, MIZ-1 was found to occupy many more sites in a MYC-dependent manner, indicating a mechanistic difference between the physiological and oncogenic properties of MYC. Together, this indicates an additional layer of control, possibly a fine-tuning mechanism through which MYC can modulate the transcription of E-box containing target genes shaping transcriptional amplification (see Figure 3A). 

## 3. Recruitment of Chromatin Modifiers for MYC-Dependent Transrepression

MYC’s function as a site-specific transcription factor includes not only the activation but also the repression of genes (both protein-coding as well as noncoding RNAs). In fact, both mechanisms are essential for MYC-driven tumor initiation and maintenance [62,63], and both mechanisms depend on the recruitment of chromatin modifying co-factors. An increasing number of non-canonical target genes have been identified that MYC represses through protein-protein interactions with zinc finger transcription factors such MIZ-1 [62], SP1 [16], nuclear factor Y (NF-Y) [61], ying yang 1 (YY1) [64], and transcription factor II I (TFII-I) [65].

**Figure 4:**
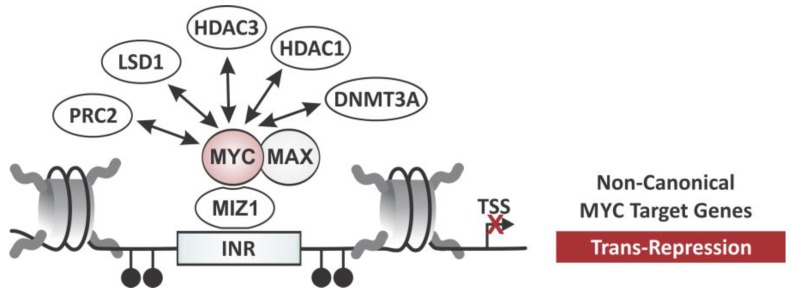
Site-specific transcriptional repression of target genes through the MYC network. MYC–MAX/MIZ-1 mediated transrepression of non-canonical target genes such as Cyclin Dependent Kinase Inhibitor 1A (*CDKN1A*/p21CIP) and Cyclin Dependent Kinase Inhibitor 2B (*CDKN2B*/p15INK4B) involves recruitment of chromatin co-repressors. DNMT3A suppresses the corresponding gene through hypermethylation of CpGs in the vicinity. Recruitment of HDAC1 and HDAC3 contribute to histone deacetylation and thus silencing of certain genes. The demethylase LSD1 removes active H3K4me marks contributing to gene repression. The PRC2 repressive complex also interacts with MYC to enhance gene silencing. Filled circles represent methylated CpGs; transcription start site (TSS).

The interaction with MIZ-1 is the best characterized example of how MYC represses transcription. MIZ-1 recognizes a consensus sequence known as INR in core promoters and, in the absence of MYC, activates the transcription of the corresponding gene (see Figure 4). However, MYC binding interferes with the transcriptional activator function of MIZ-1, blocking the interaction with co-activators while facilitating the recruitment of co-repressor complexes [17,66,67,68]. Evidence for MYC’s function as a repressor stems from a point mutant, MYC^V394D^, which is selectively deficient in its ability to interact with MIZ-1, while still dimerizing with MAX to transactivate canonical target genes. MYC^V394D^ is unable to repress wild-type MYC targets such as Cyclin Dependent Kinase Inhibitor 1A (*CDKN1A*/p21CIP) and Cyclin Dependent Kinase Inhibitor 2B (*CDKN2B*/p15INK4B), in a transgenic lymphoma model [69]. Furthermore, protein-protein binding assays suggest that MYC displaces co-activators such as p300/CBP from interacting with MIZ-1 [17]. This switch in transcriptional activity can be explained by MIZ-1 playing a role in preventing the association between MYC and the co-activator p300, resulting in a decrease of histone acetylation. 

### 3.1. MYC-Dependent Transrepression via DNA Methyltransferases

In addition to the displacement of co-activators, MYC-mediated gene repression is associated with the recruitment of co-repressors. MYC–MAX/MIZ-1 complexes have been shown to recruit the de novo DNA methyltransferase 3a (DNMT3A) to non-canonical targets such as *CDKN1A* (p21CIP) and *CDKN2B* (p15INK4B) [70], increasing the DNA methylation of promoter regions or nearby CpG islands [70]. This is important during tumorigenesis, where MYC suppresses cell cycle dependent kinase inhibitors (CDKIs) to antagonize differentiation and cellular senescence, instead promoting cell proliferation [17,66,69]. There is some evidence that MYC similarly interacts with DNMT3B [70]. In non-small-cell lung cancer (NSCLC), MYC recruits DNMT3B to the promoter of the tumor suppressor, Ras Association Domain-containing Protein 1 (*RASSF1A*), silencing its expression through DNA hypermethylation [71,72]. The recruitment of DNMTs is an attractive concept, since it provides an explanation for the aberrant DNA methylation pattern observed for tumor suppressor genes in human tumors. Whether transrepression by MYC–MAX/MIZ-1 during tumorigenesis generally requires DNMT3A and DNMT3B, and whether this applies also to physiological conditions is less understood. Nonetheless, the implication that DNMTs are recruited by MYC to generate specific DNA methylation patterns within a cell is intriguing, specifically in the light of DNMT inhibitors being exploited for therapeutic purposes [73,74,75,76].

### 3.2. MYC-Dependent Transrepression via Histone Deacetylases

While the role of histone acetylation in MYC-dependent transcriptional repression is less well characterized, two HDACs, HDAC1 [77,78,79] and HDAC3, have been reported to interact with MYC. In both cases, they have been identified as part of co-repressor complexes recruited by MYC to suppress transcription. HDAC1 was found to be recruited to the promoter of tissue transglutaminase (*tTG*) [79] and HDAC3 to the promoter of Inhibitor of DNA Binding 2 (*ID-2*) and Growth Arrest and DNA Damage-Inducible Protein GADD153 (*GADD153*) [80,81], correlating with a decrease in histone acetylation in these loci and transrepression of the corresponding genes. It would be interesting to see whether this represents a general mechanism for all INR-regulated or even all MYC-repressed genes. Support for the latter concept comes from the finding that MYC also exploits HDAC3 for transrepression of microRNAs (*MIR-15A/MIR-16-1* and *MIR-29*). The fact that those microRNAs have tumor-suppressive function in turn strengthens the notion that transrepression of genes is critical for MYC-dependent transformation [82]. In the classic model, MYC’s ability to recruit either HATs or HDACs depends on the DNA binding motif. MYC–MAX complexes interact with HATs for transactivation of canonical target genes, while MYC–MAX/MIZ-1 recruits HDACs for transrepression of non-canonical target genes. However, whether the recruitment of HDACs extends to MYC–MAX/MIZ-1 complexes repressing E-box promoters remains to be shown. Nonetheless, the fact that HDAC inhibitors have already been exploited as therapeutic strategies for hematologic malignancies raises intriguing possibilities for further studies aiming at other cancer types expressing deregulated MYC [83,84,85].

### 3.3. MYC-Dependent Transrepression via Histone Demethylases

Histone KDMs are a double-edged sword in MYC-dependent transcriptional regulation, as they are involved in both repression and activation of MYC-target genes. Lysine-Specific Histone Demethylase 1A (LSD1) (also known as KDM1A and AOF2) cooperates with N-MYC to repress tumor suppressor genes, contributing to tumor maintenance in neuroblastoma [86]. LSD1 has been known to remove mono- and di-methyl groups from histone H3 lysine 4 (H3K4me2/me3) and lysine 9 (H3K9me2/me3) [87,88], but can also demethylate non-histone proteins such as p53 and E2F [89,90]. LSD1 has been isolated as a component of several co-repressor complexes including REST corepressor 1 (CoREST), C-terminal-binding protein 1 (CtBP), HDAC1, and HDAC2 [91,92], suggesting a role in transcriptional repression. Moreover, H3K4 demethylation and histone deacetylation by LSD1-containing complexes seem to be linked, both contributing to MYC’s repressive function. Proteomic analysis revealed that LSD1 directly interacts with N-MYC via MB III [86]. Moreover, LSD1 co-localizes with N-MYC at the promoter of *CDKN1A* (p21CIP) and Clusterin (*CLU*), two tumor suppressor genes, thereby functionally cooperating with N-MYC in neuroblastoma initiation and progression [86]. 

Interestingly, by interacting with different co-factors, LSD1 also seems capable of activating genes. MYC-dependent transactivation of E-box genes in rat fibroblasts has been proposed to involve LSD1-mediated demethylation of H3K4 [93]. Given the fact that H3K4 methylation is an active chromatin mark, this seems counter-intuitive at first. However, upon serum stimulation, LSD1 has been found to form a complex with MYC at the E-boxes sites of Nucleolin (*NCL*) and Carbamoyltransferase-dihydroorotase (*CAD*), causing cycles of methylation and demethylation of lysine 4 in histone H3 (H3K4me2/me3), before H4 acetylation increases [93]. These transient LSD1-mediated methylation events produces H_2_O_2_ causing the recruitment of oxidative repair enzymes to E-box genes [93]. The concept of linking histone demethylation to oxidation and DNA repair has been proposed to be implicated in the serum-induced assembly of the transcription initiation complex, although further studies are necessary to validate and generalize this mechanism. It remains to be seen whether this mechanism applies to all MYC-activated promoters, or only a specific set of MYC target genes.

### 3.4. MYC-Dependent Transrepression and Polycomb Proteins

MYC's physiologic functions are important for embryonic stem cells (ESC), and its ectopic expression contributes to the reprogramming of somatic cells into induced pluripotent stem cells (iPSC). In ESCs, MYC is thought to fulfill two important functions, contributing to self-renewal through maintaining an undifferentiated state and promoting cell cycle progression. While the exact molecular mechanism of how MYC regulates stemness has not yet been fully elucidated yet, MYC has been found to regulate the expression and the recruitment of Polycomb Repressive Complex 2 (PRC2) proteins, providing a mechanism how it suppresses developmental genes in ESCs [94]. PRC2 is known as chromatin-modifying complex that implements transcriptional silencing, regulating hundreds of genes critical for development (for a review, see [95]). Its core unit is composed of Enhancer of Zeste Homolog 2 (EZH2), Suppressor of Zeste 12 (SUZ12), and Embryonic Ectoderm Development (EED). EZH2 is a HMT that catalyzes tri-methylation of histone H3 at lysine 27 (H3K27me3) [96], which is recognized by Chromobox (CBX) family proteins of the Polycomb Repressive Complex 1 (PRC1). The E3 ligase, Really Interesting New Gene 1B Protein (RING1B), then facilities mono-ubiquitination of histone H2A on K119 (H2AK119ub1) ultimately leading to RNA Pol II pausing and silencing. In addition, EZH2 serves as a scaffold for the recruitment of DNMTs to target gene promoters connecting histone methylation and DNA methylation [97].

MYC utilizes the repressive PRC2 machinery in ESCs, by increasing the transcription of all core components of PRC2 (SUZ12, EZH2, and EED) as well as PRC2-associated factors typically found in ESCs (including Jumonji and AT-Rich Interaction Domain Containing 2 (JARID2), esPRC2p48, and Metal Response Element Binding Transcription Factor 2 (MTF2) through binding E-box sites in their promoter regions [94]. Furthermore, both MYC and N-MYC directly interact with PRC2 proteins (EED and Adipocyte Enhancer-Binding Protein 2 (AEBP2)) through the MB II domain [98]. Together, this indicates a MYC-driven positive feedback loop maintaining an undifferentiated state through chromatin-mediated repression of lineage-specific gene expression programs.

MYC and PRC2 cooperate not only in ESCs, but also during tumorigenesis; however, the role of PRC2 in cancer is more complex, as its function depends on the tissue context. Loss-of-function mutations in EZH2, EED, and SUZ12 have been reported for T-cell acute lymphoblastic leukemia (T-ALL) and MDS [99,100], suggesting a role as tumor suppressors in specific hematological malignancies. This is supported by the finding that PRC2 acts as a tumor suppressor in *Eμ-myc*-driven B-cell lymphoma by restricting self-renewal capabilities [101]. In contrast, EZH2 functions as a tumor promoter in N-MYC-driven neuroblastoma. It directly interacts with N-MYC, leading to the repression of the putative tumor suppressor gene *CLU* [102]. Furthermore, EZH2 and SUZ12 interact with N-MYC through MB III in castration-resistant prostate carcinoma (CRPC), leading to abrogation of androgen receptor signaling and consequently driving progression to neuroendocrine prostate cancer (NEPC), the aggressive subgroup of late-stage prostate cancer [103]. Together, this indicates that N-MYC cooperates with EZH2 to drive the neuroendocrine phenotype in prostate cancer, thereby providing a rationale for therapeutic strategies targeting EZH2 [103].

### 3.5. MYC-Dependent Transrepression and ATP-Dependent Chromatin Remodeling

Another class of co-repressors that is recruited by MYC are 48 KDa TBP-Interacting Protein (TIP48)/49 KDa TBP-Interacting Protein (TIP49) [104], which function as ATPase/helicase rather than histone modifiers. The dependence on their interaction with the MB II domain of MYC already provides a hint for biological activity, including blocking differentiation and oncogenic transformation. Indeed, it has been demonstrated that TIP48 and TIP49 are essential co-factors for MYC-driven neoplastic transformation. Furthermore, their interaction with MYC/MIZ-1 is essential for cell growth and proliferation during normal Drosophila and Xenopus development [105,106]. Even though the precise function of their relationship with MYC under physiologic conditions remains unresolved, it has been speculated that TIP48 and TIP49 bridge basic transcription machinery and sequence-specific transcription factors and act as transcriptional repressors in this context.

## 4. MYC as a Master Regulator the Cancer Epigenome and Transcriptome

**Figure 5:**
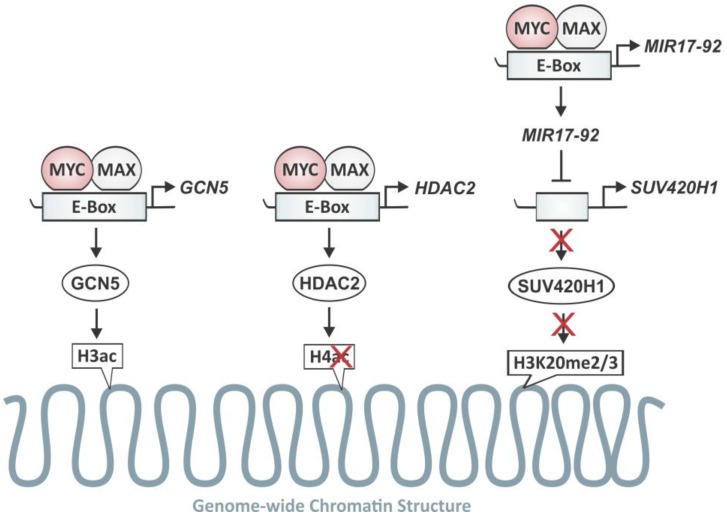
MYC as a master regulator of the cancer epigenome and transcriptome. The *MYC* oncogene deregulates histone acetylation and methylation in a global fashion with implication for the cancer epigenome and transcriptome. MYC–MAX complexes recognize an E-Box sequence in the *GCN5* promoter, leading to overexpression of GCN5. The consequently increased histone acetyltransferase (HAT) activity of GCN5 increases genome-wide acetylation of H3 and H4 (H3ac and H4ac). MYC–MAX also binds to the *HDAC2* promoter and upregulates its transcription, leading to an increase in HDAC2 activity. MYC activates the transcription of *MIR17-92*, which represses the histone methyltransferase (HMT) variegation 4-20 homolog 1 (*SUV420H1*), implicating that MYC prevents the methylation of histone H3 at K20 on a genome-wide level.

MYC’s role as a site-specific transcription factor regulating the expression of a large set of specific target genes by remodeling local chromatin structure has long been thought to be the key to its diverse cellular functions. However, a new perspective has been provided by studies that reveal that the *MYC* oncogene influences chromatin structure in a global fashion (Figure 5). These findings indicate an exciting role for MYC that extends beyond the traditional concept of a site-specific transcription factor and promises new directions for therapeutic anti-MYC strategies.

The first evidence of MYC’s global reach stem from neuronal progenitor cells in which the disruption of N-MYC expression causes widespread changes in chromatin organization, accompanied by nuclear condensation and heterochromatin formation [107]. These genome-wide changes are characterized by a marked decrease in histone H3 and H4 acetylation and an increase in H3K9me3, both indicative of gene silencing. While these observations could not be explained by the sheer number of MYC binding sites in the genome and MYC’s effect on local chromatin, the oncogene was found to influence chromatin in a genome-wide fashion through upregulation of the HAT, GCN5 [107]. N-MYC (subsequently demonstrated also for c-MYC) was found to directly bind to two E-box sequences in the *GCN5* promoter, thereby increasing its transcription in tumor cells. Consequently, GCN5 activity increased the widespread acetylation of histones, accumulating active chromatin domains. This established *GCN5* as a direct MYC target gene and provided the first evidence for regulation of genome-wide chromatin organization by an oncogene.

Subsequently, N- and c-MYC’s influence on global chromatin architecture has been demonstrated for various additional cell types [107,108]. In human B lymphocytes, serving as a Burkitt’s lymphoma model (P493-6), the suppression of a conditional c-MYC allele induces global heterochromatic regions resembling the phenotype described for N-MYC disruption in neuronal progenitor cells [107]. Similarly, the genetic inactivation of c-MYC in conditional mouse models of osteosarcoma, hepatocellular carcinoma and T-cell ALL triggered a global reduction in histone H4 acetylation and an increase in heterochromatic H3K9me3 [108]. The switchable nature of MYC in these transgenic tumor models allowed for analysis of the dynamics in chromatin structure and associated gene expression patterns. Within hours of MYC inactivation, chromatin changes become apparent and develop to widespread inactive chromatin, including senescence-associated heterochromatic foci (SAHF) [107,108]. In parallel to chromatin, changes in gene expression programs occur in a similarly time-dependent manner [109,110]. Further experiments are needed to substantiate a causative relationship between both events. Intriguingly, inactivation followed by the reactivation of MYC in a conditional osteosarcoma mouse model does not reverse the entire gene expression program controlled by MYC [109]. Furthermore, MYC’s ability to bind to promoter regions was altered, suggesting that permanent changes in the chromatin architecture affect whether certain genes are susceptible to MYC regulation. 

In parallel, MYC seems to also control histone deacetylation in a genome-wide fashion. HDAC2 expression has been found increased in a MYC-dependent fashion during APC-driven colorectal tumorigenesis [111]. Furthermore, both N-MYC and c-MYC were found to upregulate HDAC2 expression in neuroblastoma and pancreatic cancer, respectively, which contributed to MYC-induced tumor cell proliferation and blocked apoptosis in these models [112]. This depends on a MYC binding site in the *HDAC2* promoter region, which was confirmed by chromatin immunoprecipitation assay [113]. This demonstrates that MYC is capable of upregulating *HDAC2* gene expression, that MYC-induced HDAC2 overexpression contributes to MYC-induced cancer cell proliferation, and that HDAC2 is likely to be one of the key factors responsible for MYC-induced malignant transformation, tumor initiation and progression in vivo [112]. The notion that MYC upregulates the HAT, GCN5, and HDAC2 raises questions regarding their differential specificity and function on a genome-wide level.

In contrast to histone acetylation, the role of global histone methylation in regulating MYC-mediated gene expression is more complex. Even though MYC overexpression had been observed to induce both localized and widespread histone H3K4 tri-methylation (and suppressed H3K9me3), these changes were thought to be a reflection of the proliferative state of the cell rather than a direct consequence of MYC’s action. Only recently has light been shed on the mechanism by which MYC utilizes the dual function of histone methylation as an activator or repressor of transcriptome. 

MYC has been uncovered to suppresses the SIN3 Transcription Regulator Family Member B (*SIN3B*), HMG-box Transcription Factor 1 (*HBP1*), suppressor of Variegation 4–20 Homolog 1 (*SUV420H1*), and B Cell Translocation Gene 1 (*BTG1*) through *miR-17-92* [114]. SIN3B interacts with HBP1 and recruits HDACs to silence proliferation-related genes and mediate cell cycle exit and senescence [115,116,117]. SUV420H1 catalyzes di- and tri-methylation of histone H4K20 (H4K20me2 and H4K20me3) [118,119,120]. H4K20me3 is a marker for heterochromatin and cellular senescence, and is commonly lost in human cancers [121]. Both H4K20me2 and H4K20me3 were found to increase upon MYC inactivation in lymphoma [114]. BTG1 is a tumor suppressor that is frequently lost in ALL [122,123] and is known to activate the HMT, PRMT1, to di-methylate histone H4 arginine 3 (H4R3me2) [124,125]. H4R3me2 is increased upon MYC inactivation in lymphoma [114]. Once reactivated, these chromatin modifiers promote cell cycle arrest and cellular senescence, supporting the notion that MYC’s ability to sustain autonomous proliferation, self-renewal, and survival is mediated through chromatin regulatory and survival switches.

The shutoff of this epigenetic switch contributes to *MYC* oncogene addiction, which is antagonized by Transforming Growth Factor β (TGF-β) and SMADs. The TGF-β/SMAD pathway can have tumor promoting or suppressing functions depending on the context (reviewed in [126]). Hence, there has been great interest in TGF-β mediated processes in cancer with the goal of providing therapeutic strategies. During lymphomagenesis TGF-β counteracts MYC’s function by inducing the HMT, SUV39H1, responsible for inactive H3K9me3 resulting in cellular senescence in the absence of MYC [127]. SUV39H1 deficiency results in loss of H3K9me3, increased genomic instability, and the development of B- and T- cell lymphomas in mice, indicating its important role for preventing malignant transformation [127]. However, there is no evidence that MYC directly regulates *SUV39H1*, but it is speculated that MYC indirectly suppresses *SUV39H1* through TGF-β to drive cellular proliferation [128,129].

Furthermore, MYC’s influence on chromatin structure extends beyond gene regulatory or genic regions and also includes expansive intergenic regions. Genomic location analyses suggest that the inactivation of N-MYC depletes large portions of active histone marks in the genome (H3K9 acetylation and H3K4 methylation) [130]. These results implicate that MYC’s ability to induce and maintain widespread regions of active chromatin is as important as its ability to modulate the transcription of individual genes. Indeed, MYC has been shown to have broader effects on chromatin remodeling. MYC changes histone localization patterns through incorporation of the H2A.Z isoform into its target promoters, allowing for activation of target genes [39]. Another example for a chromatin-associated protein, that has been identified as a direct MYC target is the insulator protein, CCCTC-Binding Factor (*CTCF*), which is thought to allow for transcriptional alterations of wide regions of the genome by blocking facultative heterochromatin [131,132]. MYC also directly regulates Metastasis-associated protein 1 (*MTA1*), a component of the nucleosome remodeling and deacetylating (NuRD) co-repressor complex [133]. Since MTA1 has been linked to epithelial to mesenchymal transition (EMT) [134], it has been speculated that MYC is involved in this process through upregulation of MTA1, ultimately leading to increased levels of the SNAIL repressor driving the EMT process. 

## 5. MYC-Driven Transcriptional Amplification

Fueling the oncogene’s enigmatic reputation further and intensifying research interest, recent reports reveal that MYC deregulates large gene expression programs in tumor cells through transcriptional amplification. When overexpressed, MYC accumulates in the promoter regions of actively transcribed genes and acts as a general amplifier, further increasing the transcriptional output, boosting a cell's gene expression profile [41]. Thus, rather than specifying the subset of genes that will be actively transcribed in any particular cell in a site-specific manner, MYC amplifies the output of the existing gene expression program. MYC facilitates transcription elongation by stimulating the recruitment of the Positive Transcription Elongation Factor b (P-TEFb) and the Mediator complex [40,135]. The large Mediator complex, an essential co-activator of RNA Pol II also involved in chromatin “looping”, which brings distant chromosomal regions into close physical proximity to each other, interacts through STAGA with amino acids 1–110 of MYC [136]. P-TEFb releases RNA Pol II from pausing by phosphorylating its C-terminal domain (CTD) [137,138,139]. MB I and II also mediate the association with other effectors of MYC activity such as Bromodomain-Containing Protein 4 (BRD4) which is also involved in P-TEFb recruitment [135,137,138]. Therefore, MYC appears to play a critical role in RNA Pol II pause release rather than RNA Pol II recruitment, revealing an important facet MYC controls to drive transcriptional amplification. Furthermore, it has been implied that MYC may be tethered to chromatin domains such as euchromatic islands (active chromatin characterized by H3K4me, H3K79me, and H3ac) and this tethering is crucial for the rate-limiting step of MYC binding to site-specific DNA consensus sequences [140]. The overall increase of transcriptional output by MYC has a dramatic effect on proliferation and cellular biogenesis. The aspect of transcriptional amplification may be able to explain rate-limiting restraints on MYC-driven proliferation and also why MYC has broad oncogenic capabilities across a number of tissues and cell types. As an alternative explanation for the genome-wide upregulation of active genes, a recent study proposes that MYC acts mainly through controlling a selective group of genes, which in turn are responsible for global amplification either co- or post-transcriptionally [141]. Together these studies support an evolving new dual model in which MYC’s influence on chromatin is far more complex than previously imagined. The ability of MYC to act both locally and globally on chromatin may be responsible for its wide-ranging effects on the biology of stem and tumor cells. However, the new dimension of MYC’s reach also raises the question of how to integrate the new global and the classic site-specific mechanisms into one unified model. This becomes specifically apparent when attempting to explain the role of co-repressor recruitment in the context of genome-wide transcriptional amplification. It has been speculated that MYC’s ability to repress genes in a site-specific manner might be a negative feedback loop, representing a control mechanism that responds to the unrestrained expression of other MYC targets. Providing a mechanistic basis for this, recent reports extend the repressive abilities of MYC–MAX/MIZ-1 to E-box containing genes indicating a fine-tuning mechanism [61]. Given the importance of MYC-mediated gene repression for tumorigenesis, it would be interesting to see whether MYC is also capable of repressing genes in a genome-wide fashion, complementing the global amplifying model by providing a mechanism to further modulate the transcriptional output of a cell.

### DNA Sequence-Specific vs. Tethered Recruitment of MYC

**Figure 6:**
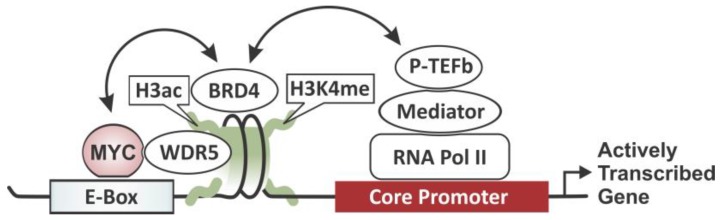
MYC-driven transcriptional amplification. MYC binds actively transcribed genes that are characterized by histone H3 acetylation (H3ac) and lysine 4 methylation (K4me). MYC binding can be facilitated either through E-box elements and/or tethered recruitments via WDR5. In turn Bromodomain-Containing Protein 4 (BDR4) is recruited, which stimulates the Positive Transcription Elongation Factor b (P-TEFb) via a Mediator complex to trigger RNA Polymerase II (RNA Pol II) pause release.

The classic model of MYC as a sequence-specific transcription factor leaves important questions unanswered. It is unable to explain the diversity of transcriptional responses MYC provokes in different cell types, indicating an inherent plasticity in MYC’s selection of target genes. Hence, additional mechanisms must exist that determine which genomic loci are occupied by MYC in a given cell type. It has been speculated that the binding of MYC to its target genes is not solely determined by a specific DNA sequence, but that MYC binding site recognition depends on chromatin structure. Early clues came from large-scale location analyses revealing that the active H3 lysine 4 (K4) and 79 (K79) methylation marks are a strict prerequisite for recognition of any target site by MYC, suggesting that distinct histone variants or modifications can modulate protein recognition [15,140]. This bias for MYC to bind chromatin enriched for active histone modifications was substantiated by several genome-wide location studies [41,61,141]. More recent reports challenge the classic model further, proposing a specific mechanism for a tethered recruitment via WD Repeat Domain 5 (WDR5) [142] (see Figure 6). WDR5 is a highly conserved chromatin “reader” found in multiple protein complexes [143], including the Myeloid/Lymphoid or Mixed-Lineage Leukemia (MLL) histone methyltransferases that catalyze H3K4 methylation and the histone demethylase LSD1 that removes H3K4/K9 marks [144]. Genome-wide location analyses reveal that MYC and WDR5 colocalize extensively on active chromatin, with approximately 80% of the genomic loci occupied by MYC also bound by WDR5 [142]. WDR5 was found to directly interact with MYC via the MB III motif in the central region [145,146], suggesting a scenario in which WDR5 mediates the recruitment of MYC to active chromatin loci. However, this model still needs to be challenged and several important questions remain to be answered. For example, how is WDR5 itself tethered to chromatin, and is MYC competing with other WDR5-binding partners such as MLL? Characterizing the MYC–WDR5 complex biochemically to identify other proteins that associate with MYC through WDR5 should provide valuable information about the molecular context in which the two proteins interact with chromatin. Further studies will be necessary to unravel the specific details, but the above results suggest an intriguing role of the WDR5–MYC interaction in tethering MYC to chromatin and indicate a critical function of WDR5 for MYC-driven tumorigenesis.

## 6. Therapeutic Strategies: Targeting Epigenetic Mechanisms in MYC-Positive Cancers

**Figure 7:**
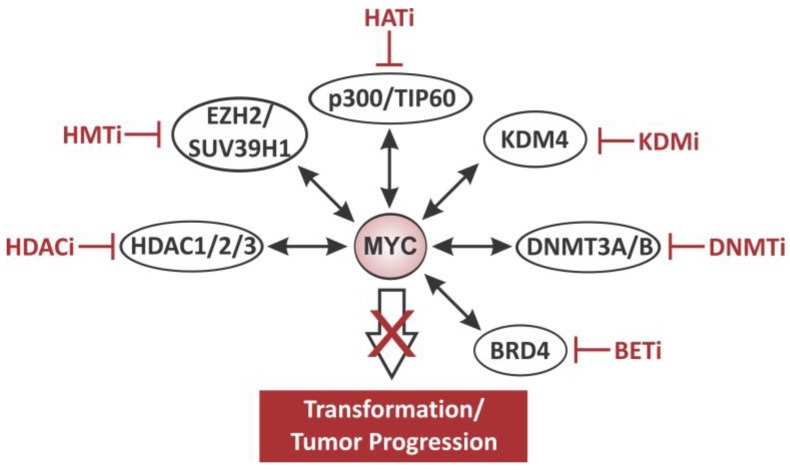
Targeting the MYC network with epigenetic inhibitors for therapeutic purposes. Schematic representation of different therapeutic strategies to target the MYC network through chromatin-modifying co-factors: Histone deacetylase inhibitors (HDACi), histone methyltransferase inhibitors (HMTi), histone acetyltransferase inhibitors (HATi), lysine-specific histone demethylase inhibitors (KDMi), DNA methyltransferase inhibitors (DNMTi), and Bromodomain and Extra-Terminal motif inhibitors (BETi).

The notion that inactivation of MYC can cause tumor regression in animal models by eliciting oncogene addiction, makes it an attractive target for therapeutic anti-cancer strategies. However, despite considerable efforts in that regard, a pharmacologic inhibitor that directly targets MYC and can be used to translate these findings into the clinical setting remains elusive. Recently, novel therapeutic strategies have emerged that promise to take advantage of the reversibility of chromatin modifications to adjust the epigenome in ways that slow cell proliferation and induce cellular senescence or apoptosis in MYC-deregulated cancers. Targeting MYC through chromatin associated co-factors that affect either the expression and/or the function of the oncogene has proven to be effective in various cell lines and animal models (for an overview, see Figure 7). Despite the fact that these strategies can cause off-target effects, some have yielded highly promising results and are tested in clinical trials, paving the road for epigenetic drugs to treat MYC-associated human cancers.

### 6.1. DNA Methyltransferase Inhibitors (DNMTi)

One of the first epigenetic therapeutics brought about and used were the DNA demethylation agents 5-aza-cytidine (azacytidine) and its analog 5-aza-2′-deoxycytidine (decitabine) [75,76]. Azacytidine and decitabine act by inserting nucleoside analogs into DNA and thus inhibiting binding and enzymatic function of DNMTs, resulting in progressive demethylation throughout cell divisions [73,74]. Low dosages of these inhibitors have been approved by the FDA for use in disorders such as myelodysplastic syndromes (MDS) [147]. Both agents can prolong survival and reduce the time it takes for MDS to progresses into ALL or acute myeloid leukemia (AML) [148,149,150,151]. Interestingly, decitabine was able to suppress *Eµ*-driven translocated MYC through upregulation of NF-ĸB and ID2 and thereby inhibit cell proliferation in Burkitt’s lymphoma [152]. Azacytidine and decitabine might be promising therapeutic strategies for translocated MYC, found not only in Burkitt’s lymphoma, but also in some diffuse large B-cell lymphoma (DLBCL) and other types of non-Hodgkin lymphoma [153,154]. Despite promising features, the downside of broad demethylation agents is their cytotoxicity when administered in high doses and the lack of cell type specificity, resulting in off-target effects. Moreover, DNA methylation is a double-edged sword in tumorigenesis and loss of function of specific DNMTs can accelerate the onset of cancer in certain circumstances [155]. This provides an avenue of exploration into inhibitors of specific DNMTs, such as DNMT1, DNMT3A, and DNMT3B, which may prove to be more beneficial than broad demethylating agents like azacytidine and decitabine.

### 6.2. Histone Acetyltransferases Inhibitors (HATi)

Acetylation of nucleosomal histones is a key mechanism through which MYC facilitates transactivation of genes important for tumor development and progression. Consequently, histone acetyltransferases inhibitors have been explored as therapeutic strategy for MYC-associated tumors. Broadly acting HAT inhibitors such as PU139 and PU141 decreased proliferation and activated apoptosis in vitro in various cancer types including ovarian carcinoma, hepatocellular carcinoma, and colon adenocarcinoma through blocking GCN5, p300/CBP, and cAMP-Responsive Element-Binding Protein 1 (CREB) activity. Intriguingly, both inhibitors also affected growth of neuroblastoma in xenograft models [156]. However, the underlying mechanism, whether decreased histone acetylation effect gene expression programs critical for proliferation or activate fail-safe mechanisms such as senescence or apoptosis, remains unclear. More specific inhibitors for HATs have begun to emerge, such as small molecules that target TIP60. TIP60 inhibitors were efficacious in castrate-resistant prostate cancer and breast cancer in reducing proliferation and inducing apoptosis; however, to our knowledge TIP60 inhibition has not yet been applied as a therapeutic strategy for cancers that are specifically MYC-addicted [157,158]. 

The function of p300/CBP has been linked to the development of various human cancers, including solid tumors and hematological malignancies. p300/CBP is critical for MYC to drive cell proliferation [159]; however, p300/CBP interacts with upwards of 400 different proteins and is involved in multiple cellular processes, making it difficult to target the MYC–p300 interaction specifically. While many experimental p300 inhibitors have been explored, the most potent and specific inhibitor described so far is C646. C646 caused suppression of MYC expression and apoptosis in CBP-deficient cancer types, specifically in CBP-mutant non-small cell lung cancer, T-ALL, and follicular B-cell lymphoma [160]. Even though downregulation of MYC transcription may be the main mechanism, rather than specifically affecting how MYC regulates its target genes, p300 might be a promising therapeutic target. However, p300 can also act as a tumor suppressor, as engineered loss of p300 in mice accelerated leukemogenesis and enhanced the transition from MDS to AML [161]. Hence, targeting p300 can be effective but it seems to be context-dependent and further studies are needed to unravel the full possibilities of p300 inhibitors in MYC-positive cancers as well as their translation into the clinical setting. In addition, a drug that directly affects the MYC–p300 interaction without affecting p300’s interaction with other proteins will be challenging to create.

### 6.3. Histone Deacetylase Inhibitors (HDACi)

While inhibiting MYC-associated HAT activity aims at antagonizing MYC-driven transactivation of tumor-promoting genes, the goal of targeting HDAC activity is to release the MYC-driven transrepression of tumor suppressor genes. HDACs have been proven to have multiple functions in driving proliferation in high MYC-expressing cancers. MYC causes overexpression of HDAC2, contributing to tumor cell proliferation, while inactivating mutations in HDAC2 reduce the frequency of tumors in mice [162,163]. MYC and HDAC2 repress Cyclin G2 (*CCNG2*), which ordinarily blocks the progression of the cell cycle, in neuroblastoma and pancreatic cancer cells to drive proliferation [112]. Inhibition of HDAC2 resulted in the downregulation of MYC expression and reactivation of the tumor suppressor, p53, which contributed to apoptosis and reduced proliferation [164]. Additionally, MYC suppressed gene transcription of tissue transglutaminase (*tTG*), which normally promotes apoptosis and induces differentiation, through recruitment of HDAC1 [79]. HDAC inhibitors, such as valproic acid (VA), trichostatin A (TSA), and suberoylanilide hydroxamic acid (SAHA), have come to light as effective therapeutic strategies for cancers characterized by high MYC expression. 

Inhibitors for different classes of HDACs (primarily class I and II due to their overexpression in various cancers) have been exploited as a broad strategy to reactivate tumor suppressor genes in various cancer types [84,85,165,166,167,168]. Many HDAC inhibitors lack specificity to distinct classes of HDACs, for example SAHA and TSA inhibit the activity of all HDAC isoforms [169]. There is current understanding that certain HDACs can be targeted based on slight differences in the corresponding active site to make these molecules more specific to certain HDAC isoforms [169]. SAHA gained FDA approval for the treatment of cutaneous T-cell lymphoma in the past decade [170], paving the way for other HDAC inhibitors to translate into clinic. HDAC inhibitors are thought to exhibit anti-tumor properties by inducing tumor suppressor genes typically silenced in cancer. These inhibitors induce apoptosis, differentiation and cytoskeletal rearrangements, while inhibiting cellular proliferation, angiogenesis, and EMT [85,164,165,166,168]. A number of HDAC inhibitors are currently in clinical trials and are probably the most encouraging of the emerging epigenetic anti-cancer therapeutics for MYC-associated cancers. 

### 6.4. Histone Methyltransferase Inhibitors (HMTi)

Histone methyltransferases inhibitors have proven to be a successful potential therapeutic strategy. EZH2, a HMT contributor to PRC2, is overexpressed in a variety of human cancers including those that express high MYC levels. EZH2 overexpression contributes to colony formation, EMT, inhibition of DNA repair regulators, and inactive H3K27 methylation marks [171]. The S-adenosylhomocysteine hydrolase inhibitor 3-Deazaneplanocin A (DZNeP) caused a reduction in EZH2, resulting in a genome-wide decrease in H3K27 methylation and apoptosis in a variety of different cancers including breast cancer, colorectal cancer, and prostate cancer, while DZNeP left normal cells unaffected; conversely, DZNeP also decreased the methylation of many other histone residues [171,172,173]. EZH2 proves to be a good therapeutic target in MYC-positive cancers; however, DZNeP lacks the needed specificity to be a promising strategy in clinical applications [172,173]. More recently, a more specific inhibitor of EZH2, GSK126, has been highly effective in treating DLBCL and has emerged as a promising therapy for translation into the clinical setting [174].

Loss of another HMT, SUV39H1, in MYC-driven myeloid leukemia prevented chromosomal instability in mouse models, indicating that it may be a potentially useful therapeutic target [175]. NV10, a small molecule inhibitor of SUV39H1, exhibited anti-cancer activity in vivo and in vitro in several cancers [176]. Specifically, NV10 was selective in targeting only pancreatic cancer cells while leaving normal cells unaffected; furthermore, it decreased the tumor volume in melanoma and lung cancer, showing promise for transition into clinic. Further studies are needed to verify if these findings also translate into MYC-positive cancers. 

### 6.5. Histone Demethylases Inhibitors (KDMi)

Similar to targeting HATs or HDACs, the reasoning for inhibiting MYC-associated HMT and KDM activity is based on antagonizing either MYC’s transactivating or transrepressing capabilities. KDMs, particularly those that remove H3K4 and H3K27 methylation are often mutated in cancer, indicating potential therapeutic targets for these enzymes. Histone demethylase inhibitors target important KDMs in MYC-expressing cancers, such as LSD1 (KDM1) and KDM4. H3K4me2 indicates active genes and LSD1 is responsible for removing mono- and di-methylated H3K4 and H3K9 marks. Therapeutic potential arose when it was discovered that the silencing of LSD1 reduced the transcription of MYC-target genes such as *NCL* [93]. Inhibitors of LSD, biguanide and bisguanidine polyamine analogs, could effectively reactivate silenced genes in colorectal cancer; furthermore, LSD inhibitors have shown promise in MLL patients through inducing differentiation [177,178]. Some LSD inhibitors, ORY-1001, Tranylcypromine, and GSK9552, have reached clinical trials but are pending results for different cancers. Pre-clinical results show exciting results for LSD inhibitors in AML, providing a good possibility of translating into clinic [179,180].

The H3K9 demethylase, KDM4, has shown promise as a therapeutic target due to the discovery that KDM4B knockout in MYC-driven neuroblastoma cells resulted in downregulation of important MYC target genes, such as *MIR17HG*, *CDC25A*, *SOX2*, *KITLG*, *VCAN*, and *SDC1* [44]. Inhibition of KDM4 via a small molecule inhibitor, SD70, diminished the onset of MLL leukemia, extended survival, exhibited low toxicity and was well tolerated in both mouse and humanized models, implicating it as a promising therapeutic strategy in AML [181]. Another promising inhibitor, NCDM-32B, slowed proliferation, decreased cell viability, and decreased EMT phenotype in basal breast cancer cells. However, NCDM-32B not only inhibited the activity of KDM4 demethylases, but also downregulated gene expression of several pathways related to proliferation and neoplastic transformation, as well as downregulation of the *MET* oncogene [182]. Because of the close homology within the KDM family, identifying small molecule inhibitors that are specific to single KDMs has been challenging and these drugs often have off-target effects. Moving forward, HDMs are highly promising for therapeutic targets for MYC-associated cancers but more specific drugs are needed to be developed to overcome the off-target effects and make these drugs tumor-cell-specific.

### 6.6. Targeting Epigenetic Readers

More recently, strategies targeting epigenetic “readers” in cancers that harbor MYC overexpression have received much attention. The discovery of super enhancers and their role in tumorigenesis led to the emergence of BET bromodomain inhibitors (BETi) as a therapeutic strategy for various cancer types [183]. BETi, such as JQ1, I-BET151, iBET762, and MS417, block the binding of BRD to acetylated lysine residues on histones, preventing RNA Pol II-mediated transcriptional elongation [184]. Super-enhancers are often associated with oncogenes such as *MYC*, and are more susceptible to BET bromodomain inhibition [183]. BET inhibition by JQ1 blocked BRD4 binding and caused a reduction in MYC expression and consequently in its target genes in multiple myeloma (MM). JQ1 suppressed proliferation through G1 cell cycle arrest and caused tumor regression and cellular senescence, presenting BETi as a successful strategy to target MYC [185,186,187]. However, despite the promise BETi show in MYC-positive cancers, these drugs have weak specificity as they downregulate hundreds of other genes, including genes not associated with super-enhancers. To be applicable in the clinic setting newly emerging molecules need to overcome the high cytotoxicity that the current generation of BETi displays in vivo [188,189]. Combination epigenetic therapy with BETi has shown promising synergistic effects, such as the cytotoxic activity of BETi, RVX2135, with the HDAC inhibitors SAHA or LBH-589 in MYC-induced lymphoma in mice [83].

Due to the role of WDR5 in recruiting MYC to E-boxes and in driving DNA replication, the potential of inhibiting WDR5 for therapy has been explored. Recent reports indicate that WDR5 is needed for proliferation and DNA replication. Two available WDR5 inhibitors with low efficacy, OICR-9429 and MM-401, show that WDR5 inhibition slowed proliferation in pancreatic cancer [190]. OICR-9429 antagonizes the WDR5–MLL interaction, selectively inhibited proliferation, and induced differentiation in human AML cells [191]; in contrast, MM-401 targets the MLL1 H3K4 methyltransferase activity and was able to inhibit MLL1 activity by blocking MLL1–WDR5 interaction and complex assembly, specifically blocking proliferation of MLL cells by inducing cell-cycle arrest, apoptosis, and myeloid differentiation without general toxicity to normal bone marrow cells or non-MLL cells [192]. In this manner, WDR5 is a highly promising therapeutic target; however, more potent and selective therapeutic options are needed. Specifically targeting the MYC–WDR5 interaction will be challenging due to the WDR5 structural binding pocket that binds MYC in the same region that WDR5 utilizes to interact with other proteins. 

Thus far, targeting chromatin modifiers associated with MYC’s oncogenic function in cancer has proven successful and effective; however, there is still much progress to be made to improve inhibitors as well as clinical efficacy for patients. Targeted strategies against tumor-cell-specific proteins hold the promise to reduce cytotoxic effects on normal tissue. The difficulty of designing small molecules that block protein–protein interactions between MYC and distinct co-factors lies in the complexity of MYC being a central hub in tumorigenesis and its co-factors’ interactions with other effector molecules. Regardless, the use of chromatin modifying drugs targeting MYC, either through directly affecting MYC expression or through affecting the cofactors MYC requires for its function, can provide an avenue for exploration into promising therapeutic strategies.

## 7. Future Perspectives

It has become evident that MYC’s ability to deregulate a wide variety of cellular processes culminating in neoplastic transformation cannot be attributed to one single trait, but instead is the product of multiple molecular mechanisms. MYC acts as a site-specific transcription factor on selective target genes by recruiting chromatin-modifying co-factors that remodel chromatin structure in the vicinity of its binding sites. However, MYC’s role as global regulator of the epigenome and transcriptome extends its function beyond the traditional concept of a classic transcription factor. MYC has been found to control the expression of an increasing number of epigenetic modifiers that facilitate widespread changes in chromatin architecture. The resulting epigenetic landscape provides the framework for MYC as a global transcriptional amplifier, boosting transcription of already active genes. 

MYC would not be MYC if there were not questions that remain unanswered. There is the chicken or the egg causality dilemma: What came first, MYC or open chromatin? Is a preexisting epigenetic state the prerequisite for MYC to further remodel the epigenome to a cancer-cell-specific pattern? Despite elusive answers to these mechanistic questions, there is strong evidence that MYC is a highly promising and effective therapeutic target. Unfortunately, MYC has also been deemed “undruggable” in clinic, so alternative strategies are needed. Targeting MYC indirectly via key chromatin modifiers in its network, taking advantage of the reversibility of epigenetic marks, provides an attractive new approach. Last but not least, one thing remains the same, regardless of the efforts over the years to understand the mechanisms underlying MYC’s neoplastic properties: the oncogene seems to preserve its enigmatic reputation.

## References

[1] Dang C.V. (2012). Myc on the path to cancer. Cell.

[2] Adhikary S., Eilers M. (2005). Transcriptional regulation and transformation by myc proteins. Nat. Rev. Mol. Cell Biol..

[3] Kress T.R., Sabo A., Amati B. (2015). Myc: Connecting selective transcriptional control to global rna production. Nat. Rev. Cancer.

[4] Blackwood E.M., Eisenman R.N. (1991). Max: A helix-loop-helix zipper protein that forms a sequence-specific DNA-binding complex with myc. Science.

[5] Little C.D., Nau M.M., Carney D.N., Gazdar A.F., Minna J.D. (1983). Amplification and expression of the c-myc oncogene in human lung cancer cell lines. Nature.

[6] Nau M.M., Brooks B.J., Battey J., Sausville E., Gazdar A.F., Kirsch I.R., McBride O.W., Bertness V., Hollis G.F., Minna J.D. (1985). L-myc, a new myc-related gene amplified and expressed in human small cell lung cancer. Nature.

[7] Schwab M., Alitalo K., Klempnauer K.H., Varmus H.E., Bishop J.M., Gilbert F., Brodeur G., Goldstein M., Trent J. (1983). Amplified DNA with limited homology to myc cellular oncogene is shared by human neuroblastoma cell lines and a neuroblastoma tumour. Nature.

[8] Dalla-Favera R., Wong-Staal F., Gallo R.C. (1982). Onc gene amplification in promyelocytic leukaemia cell line hl-60 and primary leukaemic cells of the same patient. Nature.

[9] Felsher D.W., Bishop J.M. (1999). Reversible tumorigenesis by myc in hematopoietic lineages. Mol. Cell.

[10] Evan G.I., Vousden K.H. (2001). Proliferation, cell cycle and apoptosis in cancer. Nature.

[11] Blackwell T.K., Kretzner L., Blackwood E.M., Eisenman R.N., Weintraub H. (1990). Sequence-specific DNA binding by the c-myc protein. Science.

[12] Amati B., Brooks M.W., Levy N., Littlewood T.D., Evan G.I., Land H. (1993). Oncogenic activity of the c-myc protein requires dimerization with max. Cell.

[13] Kretzner L., Blackwood E.M., Eisenman R.N. (1992). Myc and max proteins possess distinct transcriptional activities. Nature.

[14] Zeller K.I., Jegga A.G., Aronow B.J., O'Donnell K.A., Dang C.V. (2003). An integrated database of genes responsive to the myc oncogenic transcription factor: Identification of direct genomic targets. Genome Biol..

[15] Zeller K.I., Zhao X., Lee C.W., Chiu K.P., Yao F., Yustein J.T., Ooi H.S., Orlov Y.L., Shahab A., Yong H.C. (2006). Global mapping of c-myc binding sites and target gene networks in human b cells. Proc. Natl. Acad. Sci. USA.

[16] Gartel A.L., Ye X., Goufman E., Shianov P., Hay N., Najmabadi F., Tyner A.L. (2001). Myc represses the p21(waf1/cip1) promoter and interacts with sp1/sp3. Proc. Natl. Acad. Sci. USA.

[17] Staller P., Peukert K., Kiermaier A., Seoane J., Lukas J., Karsunky H., Moroy T., Bartek J., Massague J., Hanel F. (2001). Repression of p15ink4b expression by myc through association with miz-1. Nat. Cell Biol..

[18] Seoane J., Pouponnot C., Staller P., Schader M., Eilers M., Massague J. (2001). Tgfbeta influences myc, miz-1 and smad to control the cdk inhibitor p15ink4b. Nat. Cell Biol..

[19] Fernandez P.C., Frank S.R., Wang L., Schroeder M., Liu S., Greene J., Cocito A., Amati B. (2003). Genomic targets of the human c-myc protein. Genes Dev..

[20] Nair S.K., Burley S.K. (2003). X-ray structures of myc-max and mad-max recognizing DNA. Molecular bases of regulation by proto-oncogenic transcription factors. Cell.

[21] Pettersen E.F., Goddard T.D., Huang C.C., Couch G.S., Greenblatt D.M., Meng E.C., Ferrin T.E. (2004). Ucsf chimera--a visualization system for exploratory research and analysis. J. Comput. Chem..

[22] Helander S., Montecchio M., Pilstal R., Su Y., Kuruvilla J., Elven M., Ziauddin J.M., Anandapadamanaban M., Cristobal S., Lundstrom P. (2015). Pre-anchoring of pin1 to unphosphorylated c-myc in a fuzzy complex regulates c-myc activity. Structure.

[23] Tu W.B., Helander S., Pilstal R., Hickman K.A., Lourenco C., Jurisica I., Raught B., Wallner B., Sunnerhagen M., Penn L.Z. (2015). Myc and its interactors take shape. Biochim. Biophys. Acta.

[24] Herbst A., Hemann M.T., Tworkowski K.A., Salghetti S.E., Lowe S.W., Tansey W.P. (2005). A conserved element in myc that negatively regulates its proapoptotic activity. EMBO Rep..

[25] Herbst A., Salghetti S.E., Kim S.Y., Tansey W.P. (2004). Multiple cell-type-specific elements regulate myc protein stability. Oncogene.

[26] McMahon S.B., Van Buskirk H.A., Dugan K.A., Copeland T.D., Cole M.D. (1998). The novel atm-related protein trrap is an essential cofactor for the c-myc and e2f oncoproteins. Cell.

[27] McMahon S.B., Wood M.A., Cole M.D. (2000). The essential cofactor trrap recruits the histone acetyltransferase hgcn5 to c-myc. Mol. Cell Biol..

[28] Frank S.R., Parisi T., Taubert S., Fernandez P., Fuchs M., Chan H.M., Livingston D.M., Amati B. (2003). Myc recruits the tip60 histone acetyltransferase complex to chromatin. EMBO Rep..

[29] Fuchs M., Gerber J., Drapkin R., Sif S., Ikura T., Ogryzko V., Lane W.S., Nakatani Y., Livingston D.M. (2001). The p400 complex is an essential e1a transformation target. Cell.

[30] Liu X., Tesfai J., Evrard Y.A., Dent S.Y., Martinez E. (2003). C-myc transformation domain recruits the human staga complex and requires trrap and gcn5 acetylase activity for transcription activation. The J. Biol. Chem..

[31] Ciurciu A., Komonyi O., Pankotai T., Boros I.M. (2006). The drosophila histone acetyltransferase gcn5 and transcriptional adaptor ada2a are involved in nucleosomal histone h4 acetylation. Mol. Cell Biol..

[32] Kenneth N.S., Ramsbottom B.A., Gomez-Roman N., Marshall L., Cole P.A., White R.J. (2007). Trrap and gcn5 are used by c-myc to activate rna polymerase iii transcription. Proc. Natl. Acad. Sci. USA.

[33] Faiola F., Liu X., Lo S., Pan S., Zhang K., Lymar E., Farina A., Martinez E. (2005). Dual regulation of c-myc by p300 via acetylation-dependent control of myc protein turnover and coactivation of myc-induced transcription. Mol. Cell Biol..

[34] Utley R.T., Ikeda K., Grant P.A., Cote J., Steger D.J., Eberharter A., John S., Workman J.L. (1998). Transcriptional activators direct histone acetyltransferase complexes to nucleosomes. Nature.

[35] Korzus E., Torchia J., Rose D.W., Xu L., Kurokawa R., McInerney E.M., Mullen T.M., Glass C.K., Rosenfeld M.G. (1998). Transcription factor-specific requirements for coactivators and their acetyltransferase functions. Science.

[36] Vervoorts J., Luscher-Firzlaff J.M., Rottmann S., Lilischkis R., Walsemann G., Dohmann K., Austen M., Luscher B. (2003). Stimulation of c-myc transcriptional activity and acetylation by recruitment of the cofactor cbp. EMBO Rep..

[37] Zhang K., Faiola F., Martinez E. (2005). Six lysine residues on c-myc are direct substrates for acetylation by p300. Biochem. Biophys. Res. Commun..

[38] Patel J.H., Du Y., Ard P.G., Phillips C., Carella B., Chen C.J., Rakowski C., Chatterjee C., Lieberman P.M., Lane W.S. (2004). The c-myc oncoprotein is a substrate of the acetyltransferases hgcn5/pcaf and tip60. Mol. Cell Biol..

[39] Martinato F., Cesaroni M., Amati B., Guccione E. (2008). Analysis of myc-induced histone modifications on target chromatin. PLoS ONE.

[40] Rahl P.B., Lin C.Y., Seila A.C., Flynn R.A., McCuine S., Burge C.B., Sharp P.A., Young R.A. (2010). C-myc regulates transcriptional pause release. Cell.

[41] Lin C.Y., Loven J., Rahl P.B., Paranal R.M., Burge C.B., Bradner J.E., Lee T.I., Young R.A. (2012). Transcriptional amplification in tumor cells with elevated c-myc. Cell.

[42] Secombe J., Li L., Carlos L., Eisenman R.N. (2007). The trithorax group protein lid is a trimethyl histone h3k4 demethylase required for dmyc-induced cell growth. Genes Dev..

[43] Das P.P., Shao Z., Beyaz S., Apostolou E., Pinello L., De Los Angeles A., O'Brien K., Atsma J.M., Fujiwara Y., Nguyen M. (2014). Distinct and combinatorial functions of jmjd2b/kdm4b and jmjd2c/kdm4c in mouse embryonic stem cell identity. Mol. cell.

[44] Yang J., AlTahan A.M., Hu D., Wang Y., Cheng P.H., Morton C.L., Qu C., Nathwani A.C., Shohet J.M., Fotsis T. (2015). The role of histone demethylase kdm4b in myc signaling in neuroblastoma. J. Natl. Cancer Inst..

[45] Whetstine J.R., Nottke A., Lan F., Huarte M., Smolikov S., Chen Z., Spooner E., Li E., Zhang G., Colaiacovo M. (2006). Reversal of histone lysine trimethylation by the jmjd2 family of histone demethylases. Cell.

[46] Zippo A., De Robertis A., Serafini R., Oliviero S. (2007). Pim1-dependent phosphorylation of histone h3 at serine 10 is required for myc-dependent transcriptional activation and oncogenic transformation. Nat. Cell Biol..

[47] Ivaldi M.S., Karam C.S., Corces V.G. (2007). Phosphorylation of histone h3 at ser10 facilitates rna polymerase ii release from promoter-proximal pausing in drosophila. Genes Dev..

[48] Zhang Y., Wang Z., Li X., Magnuson N.S. (2008). Pim kinase-dependent inhibition of c-myc degradation. Oncogene.

[49] Sears R., Nuckolls F., Haura E., Taya Y., Tamai K., Nevins J.R. (2000). Multiple ras-dependent phosphorylation pathways regulate myc protein stability. Genes Dev..

[50] Yeh E., Cunningham M., Arnold H., Chasse D., Monteith T., Ivaldi G., Hahn W.C., Stukenberg P.T., Shenolikar S., Uchida T. (2004). A signalling pathway controlling c-myc degradation that impacts oncogenic transformation of human cells. Nat. Cell Biol..

[51] Wang J., Kim J., Roh M., Franco O.E., Hayward S.W., Wills M.L., Abdulkadir S.A. (2010). Pim1 kinase synergizes with c-myc to induce advanced prostate carcinoma. Oncogene.

[52] Kim J., Roh M., Abdulkadir S.A. (2010). Pim1 promotes human prostate cancer cell tumorigenicity and c-myc transcriptional activity. BMC Cancer.

[53] Horiuchi D., Camarda R., Zhou A.Y., Yau C., Momcilovic O., Balakrishnan S., Corella A.N., Eyob H., Kessenbrock K., Lawson D.A. (2016). Pim1 kinase inhibition as a targeted therapy against triple-negative breast tumors with elevated myc expression. Nat. Med..

[54] Cheng S.W., Davies K.P., Yung E., Beltran R.J., Yu J., Kalpana G.V. (1999). C-myc interacts with ini1/hsnf5 and requires the swi/snf complex for transactivation function. Nat. Genet..

[55] Stojanova A., Tu W.B., Ponzielli R., Kotlyar M., Chan P.K., Boutros P.C., Khosravi F., Jurisica I., Raught B., Penn L.Z. (2016). Myc interaction with the tumor suppressive swi/snf complex member ini1 regulates transcription and cellular transformation. Cell Cycle.

[56] Wilson B.G., Roberts C.W. (2011). Swi/snf nucleosome remodellers and cancer. Nat. Rev. Cancer.

[57] Bagnasco L., Tortolina L., Biasotti B., Castagnino N., Ponassi R., Tomati V., Nieddu E., Stier G., Malacarne D., Parodi S. (2007). Inhibition of a protein-protein interaction between ini1 and c-myc by small peptidomimetic molecules inspired by helix-1 of c-myc: Identification of a new target of potential antineoplastic interest. FASEB J..

[58] Kadoch C., Hargreaves D.C., Hodges C., Elias L., Ho L., Ranish J., Crabtree G.R. (2013). Proteomic and bioinformatic analysis of mammalian swi/snf complexes identifies extensive roles in human malignancy. Nat. Genet..

[59] Larsson L.G., Pettersson M., Oberg F., Nilsson K., Luscher B. (1994). Expression of mad, mxi1, max and c-myc during induced differentiation of hematopoietic cells: Opposite regulation of mad and c-myc. Oncogene.

[60] Ayer D.E., Kretzner L., Eisenman R.N. (1993). Mad: A heterodimeric partner for max that antagonizes myc transcriptional activity. Cell.

[61] Izumi H., Molander C., Penn L.Z., Ishisaki A., Kohno K., Funa K. (2001). Mechanism for the transcriptional repression by c-myc on pdgf beta-receptor. J. Cell Sci..

[62] Walz S., Lorenzin F., Morton J., Wiese K.E., von Eyss B., Herold S., Rycak L., Dumay-Odelot H., Karim S., Bartkuhn M. (2014). Activation and repression by oncogenic myc shape tumour-specific gene expression profiles. Nature.

[63] van de Wetering M., Sancho E., Verweij C., de Lau W., Oving I., Hurlstone A., van der Horn K., Batlle E., Coudreuse D., Haramis A.P. (2002). The beta-catenin/tcf-4 complex imposes a crypt progenitor phenotype on colorectal cancer cells. Cell.

[64] Shrivastava A., Saleque S., Kalpana G.V., Artandi S., Goff S.P., Calame K. (1993). Inhibition of transcriptional regulator yin-yang-1 by association with c-myc. Science.

[65] Roy A.L., Carruthers C., Gutjahr T., Roeder R.G. (1993). Direct role for myc in transcription initiation mediated by interactions with tfii-i. Nature.

[66] Wu S., Cetinkaya C., Munoz-Alonso M.J., von der Lehr N., Bahram F., Beuger V., Eilers M., Leon J., Larsson L.G. (2003). Myc represses differentiation-induced p21cip1 expression via miz-1-dependent interaction with the p21 core promoter. Oncogene.

[67] Wanzel M., Herold S., Eilers M. (2003). Transcriptional repression by myc. Trends Cell Biol..

[68] Kime L., Wright S.C. (2003). Mad4 is regulated by a transcriptional repressor complex that contains miz-1 and c-myc. Biochem. J..

[69] van Riggelen J., Muller J., Otto T., Beuger V., Yetil A., Choi P.S., Kosan C., Moroy T., Felsher D.W., Eilers M. (2010). The interaction between myc and miz1 is required to antagonize tgfbeta-dependent autocrine signaling during lymphoma formation and maintenance. Genes Dev..

[70] Brenner C., Deplus R., Didelot C., Loriot A., Vire E., De Smet C., Gutierrez A., Danovi D., Bernard D., Boon T. (2005). Myc represses transcription through recruitment of DNA methyltransferase corepressor. EMBO J..

[71] Palakurthy R.K., Wajapeyee N., Santra M.K., Gazin C., Lin L., Gobeil S., Green M.R. (2009). Epigenetic silencing of the rassf1a tumor suppressor gene through hoxb3-mediated induction of dnmt3b expression. Mol. Cell.

[72] Wang J., Walsh G., Liu D.D., Lee J.J., Mao L. (2006). Expression of delta dnmt3b variants and its association with promoter methylation of p16 and rassf1a in primary non-small cell lung cancer. Cancer Res..

[73] Momparler R.L., Bouchard J., Onetto N., Rivard G.E. (1984). 5-aza-2'-deoxycytidine therapy in patients with acute leukemia inhibits DNA methylation. Leuk. Res..

[74] Rivard G.E., Momparler R.L., Demers J., Benoit P., Raymond R., Lin K., Momparler L.F. (1981). Phase i study on 5-aza-2'-deoxycytidine in children with acute leukemia. Leuk. Res..

[75] Weiss A.J., Stambaugh J.E., Mastrangelo M.J., Laucius J.F., Bellet R.E. (1972). Phase i study of 5-azacytidine (nsc-102816). Cancer Chemother. Rep..

[76] Wijermans P., Lubbert M., Verhoef G., Bosly A., Ravoet C., Andre M., Ferrant A. (2000). Low-dose 5-aza-2'-deoxycytidine, a DNA hypomethylating agent, for the treatment of high-risk myelodysplastic syndrome: A multicenter phase ii study in elderly patients. J. Clin. Oncol..

[77] Matsuoka Y., Fukamachi K., Uehara N., Tsuda H., Tsubura A. (2008). Induction of a novel histone deacetylase 1/c-myc/mnt/max complex formation is implicated in parity-induced refractoriness to mammary carcinogenesis. Cancer Sci..

[78] Huerta M., Munoz R., Tapia R., Soto-Reyes E., Ramirez L., Recillas-Targa F., Gonzalez-Mariscal L., Lopez-Bayghen E. (2007). Cyclin d1 is transcriptionally down-regulated by zo-2 via an e box and the transcription factor c-myc. Mol. Biol. Cell.

[79] Liu T., Tee A.E., Porro A., Smith S.A., Dwarte T., Liu P.Y., Iraci N., Sekyere E., Haber M., Norris M.D. (2007). Activation of tissue transglutaminase transcription by histone deacetylase inhibition as a therapeutic approach for myc oncogenesis. Proc. Natl. Acad. Sci. USA.

[80] Kurland J.F., Tansey W.P. (2008). Myc-mediated transcriptional repression by recruitment of histone deacetylase. Cancer Res..

[81] Wang J., Elahi A., Ajidahun A., Clark W., Hernandez J., Achille A., Hao J.H., Seto E., Shibata D. (2014). The interplay between histone deacetylases and c-myc in the transcriptional suppression of hpp1 in colon cancer. Cancer Biol. Ther..

[82] Zhang X., Zhao X., Fiskus W., Lin J., Lwin T., Rao R., Zhang Y., Chan J.C., Fu K., Marquez V.E. (2012). Coordinated silencing of myc-mediated mir-29 by hdac3 and ezh2 as a therapeutic target of histone modification in aggressive b-cell lymphomas. Cancer Cell.

[83] Bhadury J., Nilsson L.M., Muralidharan S.V., Green L.C., Li Z., Gesner E.M., Hansen H.C., Keller U.B., McLure K.G., Nilsson J.A. (2014). Bet and hdac inhibitors induce similar genes and biological effects and synergize to kill in myc-induced murine lymphoma. Proc. Natl. Acad. Sci. USA.

[84] Gottlicher M., Minucci S., Zhu P., Kramer O.H., Schimpf A., Giavara S., Sleeman J.P., Lo Coco F., Nervi C., Pelicci P.G. (2001). Valproic acid defines a novel class of hdac inhibitors inducing differentiation of transformed cells. EMBO J..

[85] Kramer O.H., Zhu P., Ostendorff H.P., Golebiewski M., Tiefenbach J., Peters M.A., Brill B., Groner B., Bach I., Heinzel T. (2003). The histone deacetylase inhibitor valproic acid selectively induces proteasomal degradation of hdac2. EMBO J..

[86] Amente S., Milazzo G., Sorrentino M.C., Ambrosio S., Di Palo G., Lania L., Perini G., Majello B. (2015). Lysine-specific demethylase (lsd1/kdm1a) and mycn cooperatively repress tumor suppressor genes in neuroblastoma. Oncotarget.

[87] Shi Y., Lan F., Matson C., Mulligan P., Whetstine J.R., Cole P.A., Casero R.A., Shi Y. (2004). Histone demethylation mediated by the nuclear amine oxidase homolog lsd1. Cell.

[88] Metzger E., Wissmann M., Yin N., Muller J.M., Schneider R., Peters A.H., Gunther T., Buettner R., Schule R. (2005). Lsd1 demethylates repressive histone marks to promote androgen-receptor-dependent transcription. Nature.

[89] Huang J., Sengupta R., Espejo A.B., Lee M.G., Dorsey J.A., Richter M., Opravil S., Shiekhattar R., Bedford M.T., Jenuwein T. (2007). P53 is regulated by the lysine demethylase lsd1. Nature.

[90] Kontaki H., Talianidis I. (2010). Lysine methylation regulates e2f1-induced cell death. Mol. Cell.

[91] Humphrey G.W., Wang Y., Russanova V.R., Hirai T., Qin J., Nakatani Y., Howard B.H. (2001). Stable histone deacetylase complexes distinguished by the presence of sant domain proteins corest/kiaa0071 and mta-l1. J. Biol. Chem..

[92] Shi Y.J., Matson C., Lan F., Iwase S., Baba T., Shi Y. (2005). Regulation of lsd1 histone demethylase activity by its associated factors. Mol. Cell.

[93] Amente S., Bertoni A., Morano A., Lania L., Avvedimento E.V., Majello B. (2010). Lsd1-mediated demethylation of histone h3 lysine 4 triggers myc-induced transcription. Oncogene.

[94] Neri F., Zippo A., Krepelova A., Cherubini A., Rocchigiani M., Oliviero S. (2012). Myc regulates the transcription of the prc2 gene to control the expression of developmental genes in embryonic stem cells. Mol. Cell Biol..

[95] Simon J.A., Kingston R.E. (2009). Mechanisms of polycomb gene silencing: Knowns and unknowns. Nat. Rev. Mol. Cell Biol..

[96] Margueron R., Li G., Sarma K., Blais A., Zavadil J., Woodcock C.L., Dynlacht B.D., Reinberg D. (2008). Ezh1 and ezh2 maintain repressive chromatin through different mechanisms. Mol. Cell.

[97] Vire E., Brenner C., Deplus R., Blanchon L., Fraga M., Didelot C., Morey L., Van Eynde A., Bernard D., Vanderwinden J.M. (2006). The polycomb group protein ezh2 directly controls DNA methylation. Nature.

[98] Fagnocchi L., Cherubini A., Hatsuda H., Fasciani A., Mazzoleni S., Poli V., Berno V., Rossi R.L., Reinbold R., Endele M. (2016). A myc-driven self-reinforcing regulatory network maintains mouse embryonic stem cell identity. Nat. Commun..

[99] Ernst T., Chase A.J., Score J., Hidalgo-Curtis C.E., Bryant C., Jones A.V., Waghorn K., Zoi K., Ross F.M., Reiter A. (2010). Inactivating mutations of the histone methyltransferase gene ezh2 in myeloid disorders. Nat. Genet..

[100] Zhang J., Ding L., Holmfeldt L., Wu G., Heatley S.L., Payne-Turner D., Easton J., Chen X., Wang J., Rusch M. (2012). The genetic basis of early t-cell precursor acute lymphoblastic leukaemia. Nature.

[101] Lee S.C., Phipson B., Hyland C.D., Leong H.S., Allan R.S., Lun A., Hilton D.J., Nutt S.L., Blewitt M.E., Smyth G.K. (2013). Polycomb repressive complex 2 (prc2) suppresses emu-myc lymphoma. Blood.

[102] Corvetta D., Chayka O., Gherardi S., D'Acunto C.W., Cantilena S., Valli E., Piotrowska I., Perini G., Sala A. (2013). Physical interaction between mycn oncogene and polycomb repressive complex 2 (prc2) in neuroblastoma: Functional and therapeutic implications. J. Biol. Chem..

[103] Dardenne E., Beltran H., Benelli M., Gayvert K., Berger A., Puca L., Cyrta J., Sboner A., Noorzad Z., MacDonald T. (2016). N-myc induces an ezh2-mediated transcriptional program driving neuroendocrine prostate cancer. Cancer Cell.

[104] Wood M.A., McMahon S.B., Cole M.D. (2000). An atpase/helicase complex is an essential cofactor for oncogenic transformation by c-myc. Mol. Cell.

[105] Bellosta P., Hulf T., Balla Diop S., Usseglio F., Pradel J., Aragnol D., Gallant P. (2005). Myc interacts genetically with tip48/reptin and tip49/pontin to control growth and proliferation during drosophila development. Proc. Natl. Acad. Sci. USA.

[106] Etard C., Gradl D., Kunz M., Eilers M., Wedlich D. (2005). Pontin and reptin regulate cell proliferation in early xenopus embryos in collaboration with c-myc and miz-1. Mech. Dev..

[107] Knoepfler P.S., Zhang X.Y., Cheng P.F., Gafken P.R., McMahon S.B., Eisenman R.N. (2006). Myc influences global chromatin structure. EMBO J..

[108] Wu C.H., van Riggelen J., Yetil A., Fan A.C., Bachireddy P., Felsher D.W. (2007). Cellular senescence is an important mechanism of tumor regression upon c-myc inactivation. Proc. Natl. Acad. Sci. USA.

[109] Wu C.H., Sahoo D., Arvanitis C., Bradon N., Dill D.L., Felsher D.W. (2008). Combined analysis of murine and human microarrays and chip analysis reveals genes associated with the ability of myc to maintain tumorigenesis. PLoS Genet..

[110] Muller J., Samans B., van Riggelen J., Faga G., Peh K.N.R., Wei C.L., Muller H., Amati B., Felsher D., Eilers M. (2010). Tgfbeta-dependent gene expression shows that senescence correlates with abortive differentiation along several lineages in myc-induced lymphomas. Cell Cycle.

[111] Zhu P., Martin E., Mengwasser J., Schlag P., Janssen K.P., Gottlicher M. (2004). Induction of hdac2 expression upon loss of apc in colorectal tumorigenesis. Cancer Cell.

[112] Marshall G.M., Gherardi S., Xu N., Neiron Z., Trahair T., Scarlett C.J., Chang D.K., Liu P.Y., Jankowski K., Iraci N. (2010). Transcriptional upregulation of histone deacetylase 2 promotes myc-induced oncogenic effects. Oncogene.

[113] Bhandari D.R., Seo K.W., Jung J.W., Kim H.S., Yang S.R., Kang K.S. (2011). The regulatory role of c-myc on hdac2 and pcg expression in human multipotent stem cells. J. Cell. Mol. Med..

[114] Li Y., Choi P.S., Casey S.C., Dill D.L., Felsher D.W. (2014). Myc through mir-17-92 suppresses specific target genes to maintain survival, autonomous proliferation, and a neoplastic state. Cancer Cell.

[115] David G., Grandinetti K.B., Finnerty P.M., Simpson N., Chu G.C., Depinho R.A. (2008). Specific requirement of the chromatin modifier msin3b in cell cycle exit and cellular differentiation. Proc. Natl. Acad. Sci. USA.

[116] Grandinetti K.B., Jelinic P., DiMauro T., Pellegrino J., Fernandez Rodriguez R., Finnerty P.M., Ruoff R., Bardeesy N., Logan S.K., David G. (2009). Sin3b expression is required for cellular senescence and is up-regulated upon oncogenic stress. Cancer Res..

[117] Swanson K.A., Knoepfler P.S., Huang K., Kang R.S., Cowley S.M., Laherty C.D., Eisenman R.N., Radhakrishnan I. (2004). Hbp1 and mad1 repressors bind the sin3 corepressor pah2 domain with opposite helical orientations. Nat. Struct. Mol. Biol..

[118] Pesavento J.J., Yang H., Kelleher N.L., Mizzen C.A. (2008). Certain and progressive methylation of histone h4 at lysine 20 during the cell cycle. Mol. Cell Biol..

[119] Schotta G., Lachner M., Sarma K., Ebert A., Sengupta R., Reuter G., Reinberg D., Jenuwein T. (2004). A silencing pathway to induce h3-k9 and h4-k20 trimethylation at constitutive heterochromatin. Genes Dev..

[120] Schotta G., Sengupta R., Kubicek S., Malin S., Kauer M., Callen E., Celeste A., Pagani M., Opravil S., De La Rosa-Velazquez I.A. (2008). A chromatin-wide transition to h4k20 monomethylation impairs genome integrity and programmed DNA rearrangements in the mouse. Genes Dev..

[121] Fraga M.F., Ballestar E., Villar-Garea A., Boix-Chornet M., Espada J., Schotta G., Bonaldi T., Haydon C., Ropero S., Petrie K. (2005). Loss of acetylation at lys16 and trimethylation at lys20 of histone h4 is a common hallmark of human cancer. Nat. Genet..

[122] Lundin C., Hjorth L., Behrendtz M., Nordgren A., Palmqvist L., Andersen M.K., Biloglav A., Forestier E., Paulsson K., Johansson B. (2012). High frequency of btg1 deletions in acute lymphoblastic leukemia in children with down syndrome. Genes Chromosomes Cancer.

[123] Waanders E., Scheijen B., van der Meer L.T., van Reijmersdal S.V., van Emst L., Kroeze Y., Sonneveld E., Hoogerbrugge P.M., van Kessel A.G., van Leeuwen F.N. (2012). The origin and nature of tightly clustered btg1 deletions in precursor b-cell acute lymphoblastic leukemia support a model of multiclonal evolution. PLoS Genet..

[124] Berthet C., Guehenneux F., Revol V., Samarut C., Lukaszewicz A., Dehay C., Dumontet C., Magaud J.P., Rouault J.P. (2002). Interaction of prmt1 with btg/tob proteins in cell signalling: Molecular analysis and functional aspects. Genes Cells.

[125] Lin W.J., Gary J.D., Yang M.C., Clarke S., Herschman H.R. (1996). The mammalian immediate-early tis21 protein and the leukemia-associated btg1 protein interact with a protein-arginine n-methyltransferase. J. Biol. Chem..

[126] Lebrun J.J. (2012). The dual role of tgfbeta in human cancer: From tumor suppression to cancer metastasis. ISRN Mol. Biol..

[127] Reimann M., Lee S., Loddenkemper C., Dorr J.R., Tabor V., Aichele P., Stein H., Dorken B., Jenuwein T., Schmitt C.A. (2010). Tumor stroma-derived tgf-beta limits myc-driven lymphomagenesis via suv39h1-dependent senescence. Cancer Cell.

[128] Peters A.H., O'Carroll D., Scherthan H., Mechtler K., Sauer S., Schofer C., Weipoltshammer K., Pagani M., Lachner M., Kohlmaier A. (2001). Loss of the suv39h histone methyltransferases impairs mammalian heterochromatin and genome stability. Cell.

[129] Braig M., Lee S., Loddenkemper C., Rudolph C., Peters A.H., Schlegelberger B., Stein H., Dorken B., Jenuwein T., Schmitt C.A. (2005). Oncogene-induced senescence as an initial barrier in lymphoma development. Nature.

[130] Cotterman R., Jin V.X., Krig S.R., Lemen J.M., Wey A., Farnham P.J., Knoepfler P.S. (2008). N-myc regulates a widespread euchromatic program in the human genome partially independent of its role as a classical transcription factor. Cancer Res..

[131] Day L., Chau C.M., Nebozhyn M., Rennekamp A.J., Showe M., Lieberman P.M. (2007). Chromatin profiling of epstein-barr virus latency control region. J. Virol..

[132] Chau C.M., Zhang X.Y., McMahon S.B., Lieberman P.M. (2006). Regulation of epstein-barr virus latency type by the chromatin boundary factor ctcf. J. Virol..

[133] Zhang X.Y., DeSalle L.M., Patel J.H., Capobianco A.J., Yu D., Thomas-Tikhonenko A., McMahon S.B. (2005). Metastasis-associated protein 1 (mta1) is an essential downstream effector of the c-myc oncoprotein. Proc. Natl. Acad. Sci. USA.

[134] Nawa A., Nishimori K., Lin P., Maki Y., Moue K., Sawada H., Toh Y., Fumitaka K., Nicolson G.L. (2000). Tumor metastasis-associated human mta1 gene: Its deduced protein sequence, localization, and association with breast cancer cell proliferation using antisense phosphorothioate oligonucleotides. J. Cell. Biochem..

[135] Rahl P.B., Young R.A. (2014). Myc and transcription elongation. Cold Spring Harb. Perspect. Med..

[136] Liu X., Vorontchikhina M., Wang Y.L., Faiola F., Martinez E. (2008). Staga recruits mediator to the myc oncoprotein to stimulate transcription and cell proliferation. Mol. Cell Biol..

[137] Eberhardy S.R., Farnham P.J. (2002). Myc recruits p-tefb to mediate the final step in the transcriptional activation of the cad promoter. J. Biol. Chem..

[138] Kanazawa S., Soucek L., Evan G., Okamoto T., Peterlin B.M. (2003). C-myc recruits p-tefb for transcription, cellular proliferation and apoptosis. Oncogene.

[139] Eberhardy S.R., Farnham P.J. (2001). C-myc mediates activation of the cad promoter via a post-RNA polymerase ii recruitment mechanism. J. Biol. Chem..

[140] Guccione E., Martinato F., Finocchiaro G., Luzi L., Tizzoni L., Dall' Olio V., Zardo G., Nervi C., Bernard L., Amati B. (2006). Myc-binding-site recognition in the human genome is determined by chromatin context. Nat. Cell Biol..

[141] Sabo A., Kress T.R., Pelizzola M., de Pretis S., Gorski M.M., Tesi A., Morelli M.J., Bora P., Doni M., Verrecchia A. (2014). Selective transcriptional regulation by myc in cellular growth control and lymphomagenesis. Nature.

[142] Thomas L.R., Wang Q., Grieb B.C., Phan J., Foshage A.M., Sun Q., Olejniczak E.T., Clark T., Dey S., Lorey S. (2015). Interaction with wdr5 promotes target gene recognition and tumorigenesis by myc. Mol. Cell.

[143] Van Nuland R., Smits A.H., Pallaki P., Jansen P.W., Vermeulen M., Timmers H.T. (2013). Quantitative dissection and stoichiometry determination of the human set1/mll histone methyltransferase complexes. Mol. Cell Biol..

[144] Dou Y., Milne T.A., Ruthenburg A.J., Lee S., Lee J.W., Verdine G.L., Allis C.D., Roeder R.G. (2006). Regulation of mll1 h3k4 methyltransferase activity by its core components. Nat. Struct. Mol. Biol..

[145] Ullius A., Luscher-Firzlaff J., Costa I.G., Walsemann G., Forst A.H., Gusmao E.G., Kapelle K., Kleine H., Kremmer E., Vervoorts J. (2014). The interaction of myc with the trithorax protein ash2l promotes gene transcription by regulating h3k27 modification. Nucleic Acids Res..

[146] Dingar D., Kalkat M., Chan P.K., Srikumar T., Bailey S.D., Tu W.B., Coyaud E., Ponzielli R., Kolyar M., Jurisica I. (2015). Bioid identifies novel c-myc interacting partners in cultured cells and xenograft tumors. J. Proteom..

[147] Wang Y., Liu C., Guo Q.L., Yan J.Q., Zhu X.Y., Huang C.S., Zou W.Y. (2011). Intrathecal 5-azacytidine inhibits global DNA methylation and methyl-cpg-binding protein 2 expression and alleviates neuropathic pain in rats following chronic constriction injury. Brain Res..

[148] Jiang Y., Dunbar A., Gondek L.P., Mohan S., Rataul M., O'Keefe C., Sekeres M., Saunthararajah Y., Maciejewski J.P. (2009). Aberrant DNA methylation is a dominant mechanism in mds progression to aml. Blood.

[149] Kadia T.M., Thomas X.G., Dmoszynska A., Wierzbowska A., Minden M., Arthur C., Delaunay J., Ravandi F., Kantarjian H. (2015). Decitabine improves outcomes in older patients with acute myeloid leukemia and higher blast counts. Am. J. Hematol..

[150] Lubbert M., Suciu S., Hagemeijer A., Ruter B., Platzbecker U., Giagounidis A., Selleslag D., Labar B., Germing U., Salih H.R. (2016). Decitabine improves progression-free survival in older high-risk mds patients with multiple autosomal monosomies: Results of a subgroup analysis of the randomized phase iii study 06011 of the eortc leukemia cooperative group and german mds study group. Ann. Hematol..

[151] Saba H.I. (2007). Decitabine in the treatment of myelodysplastic syndromes. Ther. Clin. Risk Manag..

[152] Kantarjian H., Issa J.P., Rosenfeld C.S., Bennett J.M., Albitar M., DiPersio J., Klimek V., Slack J., de Castro C., Ravandi F. (2006). Decitabine improves patient outcomes in myelodysplastic syndromes: Results of a phase iii randomized study. Cancer.

[153] Guan H., Xie L., Klapproth K., Weitzer C.D., Wirth T., Ushmorov A. (2013). Decitabine represses translocated myc oncogene in burkitt lymphoma. J. Pathol..

[154] Yoon S.O., Jeon Y.K., Paik J.H., Kim W.Y., Kim Y.A., Kim J.E., Kim C.W. (2008). Myc translocation and an increased copy number predict poor prognosis in adult diffuse large b-cell lymphoma (dlbcl), especially in germinal centre-like b cell (gcb) type. Histopathology.

[155] Hlady R.A., Novakova S., Opavska J., Klinkebiel D., Peters S.L., Bies J., Hannah J., Iqbal J., Anderson K.M., Siebler H.M. (2012). Loss of dnmt3b function upregulates the tumor modifier ment and accelerates mouse lymphomagenesis. J. Clin. Investig..

[156] Gajer J.M., Furdas S.D., Grunder A., Gothwal M., Heinicke U., Keller K., Colland F., Fulda S., Pahl H.L., Fichtner I. (2015). Histone acetyltransferase inhibitors block neuroblastoma cell growth in vivo. Oncogenesis.

[157] Coffey K., Blackburn T.J., Cook S., Golding B.T., Griffin R.J., Hardcastle I.R., Hewitt L., Huberman K., McNeill H.V., Newell D.R. (2012). Characterisation of a tip60 specific inhibitor, nu9056, in prostate cancer. PLoS ONE.

[158] Gao C., Bourke E., Scobie M., Famme M.A., Koolmeister T., Helleday T., Eriksson L.A., Lowndes N.F., Brown J.A. (2014). Rational design and validation of a tip60 histone acetyltransferase inhibitor. Sci. Rep..

[159] Adhikary S., Marinoni F., Hock A., Hulleman E., Popov N., Beier R., Bernard S., Quarto M., Capra M., Goettig S. (2005). The ubiquitin ligase hecth9 regulates transcriptional activation by myc and is essential for tumor cell proliferation. Cell.

[160] Ogiwara H., Sasaki M., Mitachi T., Oike T., Higuchi S., Tominaga Y., Kohno T. (2016). Targeting p300 addiction in cbp-deficient cancers causes synthetic lethality by apoptotic cell death due to abrogation of myc expression. Cancer Discov..

[161] Cheng G., Liu F., Asai T., Lai F., Man N., Xu H., Chen S., Greenblatt S., Hamard P.J., Ando K. (2016). Loss of p300 accelerates mds-associated leukemogenesis. Leukemia.

[162] Hrzenjak A., Moinfar F., Kremser M.L., Strohmeier B., Staber P.B., Zatloukal K., Denk H. (2006). Valproate inhibition of histone deacetylase 2 affects differentiation and decreases proliferation of endometrial stromal sarcoma cells. Mol. Cancer Ther..

[163] Zimmermann S., Kiefer F., Prudenziati M., Spiller C., Hansen J., Floss T., Wurst W., Minucci S., Gottlicher M. (2007). Reduced body size and decreased intestinal tumor rates in hdac2-mutant mice. Cancer Res..

[164] Butler L.M., Zhou X., Xu W.S., Scher H.I., Rifkind R.A., Marks P.A., Richon V.M. (2002). The histone deacetylase inhibitor saha arrests cancer cell growth, up-regulates thioredoxin-binding protein-2, and down-regulates thioredoxin. Proc. Natl. Acad. Sci. USA.

[165] Dalla-Favera R., Martinotti S., Gallo R.C., Erikson J., Croce C.M. (1983). Translocation and rearrangements of the c-myc oncogene locus in human undifferentiated b-cell lymphomas. Science.

[166] Michaelis M., Michaelis U.R., Fleming I., Suhan T., Cinatl J., Blaheta R.A., Hoffmann K., Kotchetkov R., Busse R., Nau H. (2004). Valproic acid inhibits angiogenesis in vitro and in vivo. Mol. Pharmacol..

[167] Dai W., Wang C., Yu C., Yao J., Sun F., Teng L., Li Y. (2015). Preparation of a mixed-matrix hydrogel of vorinostat for topical administration on the rats as experimental model. Eur. J. Pharm. Sci..

[168] Yang D.H., Lee J.W., Lee J., Moon E.Y. (2014). Dynamic rearrangement of f-actin is required to maintain the antitumor effect of trichostatin a. PLoS ONE.

[169] Khan N., Jeffers M., Kumar S., Hackett C., Boldog F., Khramtsov N., Qian X., Mills E., Berghs S.C., Carey N. (2008). Determination of the class and isoform selectivity of small-molecule histone deacetylase inhibitors. Biochem. J..

[170] (2007). Vorinostat (zolinza) for cutaneous t-cell lymphoma. Med. Lett. Drugs Ther..

[171] Sarma K., Margueron R., Ivanov A., Pirrotta V., Reinberg D. (2008). Ezh2 requires phf1 to efficiently catalyze h3 lysine 27 trimethylation in vivo. Mol. Cell Biol..

[172] Miranda T.B., Cortez C.C., Yoo C.B., Liang G., Abe M., Kelly T.K., Marquez V.E., Jones P.A. (2009). Dznep is a global histone methylation inhibitor that reactivates developmental genes not silenced by DNA methylation. Mol. Cancer Ther..

[173] Tan J., Yang X., Zhuang L., Jiang X., Chen W., Lee P.L., Karuturi R.K., Tan P.B., Liu E.T., Yu Q. (2007). Pharmacologic disruption of polycomb-repressive complex 2-mediated gene repression selectively induces apoptosis in cancer cells. Genes Dev..

[174] McCabe M.T., Ott H.M., Ganji G., Korenchuk S., Thompson C., Van Aller G.S., Liu Y., Graves A.P., Della Pietra A., Diaz E. (2012). Ezh2 inhibition as a therapeutic strategy for lymphoma with ezh2-activating mutations. Nature.

[175] Vajen B., Modlich U., Schienke A., Wolf S., Skawran B., Hofmann W., Busche G., Kreipe H., Baum C., Santos-Barriopedro I. (2013). Histone methyltransferase suv39h1 deficiency prevents myc-induced chromosomal instability in murine myeloid leukemias. Genes Chromosomes Cancer.

[176] Baumann M., Dieskau A.P., Loertscher B.M., Walton M.C., Nam S., Xie J., Horne D., Overman L.E. (2015). Tricyclic analogues of epidithiodioxopiperazine alkaloids with promising in vitro and in vivo antitumor activity. Chem. Sci..

[177] Feng Z., Yao Y., Zhou C., Chen F., Wu F., Wei L., Liu W., Dong S., Redell M., Mo Q. (2016). Pharmacological inhibition of lsd1 for the treatment of mll-rearranged leukemia. J. Hematol. Oncol..

[178] Cheung N., Chan L.C., Thompson A., Cleary M.L., So C.W. (2007). Protein arginine-methyltransferase-dependent oncogenesis. Nat. Cell Biol..

[179] Fiskus W., Sharma S., Shah B., Portier B.P., Devaraj S.G., Liu K., Iyer S.P., Bearss D., Bhalla K.N. (2017). Highly effective combination of lsd1 (kdm1a) antagonist and pan-histone deacetylase inhibitor against human aml cells. Leukemia.

[180] Ishikawa Y., Gamo K., Yabuki M., Takagi S., Toyoshima K., Nakayama K., Nakayama A., Morimoto M., Miyashita H., Dairiki R. (2017). A novel lsd1 inhibitor t-3775440 disrupts gfi1b-containing complex leading to transdifferentiation and impaired growth of aml cells. Mol. Cancer Ther..

[181] Cheung N., Fung T.K., Zeisig B.B., Holmes K., Rane J.K., Mowen K.A., Finn M.G., Lenhard B., Chan L.C., So C.W. (2016). Targeting aberrant epigenetic networks mediated by prmt1 and kdm4c in acute myeloid leukemia. Cancer Cell.

[182] Ye Q., Holowatyj A., Wu J., Liu H., Zhang L., Suzuki T., Yang Z.Q. (2015). Genetic alterations of kdm4 subfamily and therapeutic effect of novel demethylase inhibitor in breast cancer. Am. J. Cancer Res..

[183] Loven J., Hoke H.A., Lin C.Y., Lau A., Orlando D.A., Vakoc C.R., Bradner J.E., Lee T.I., Young R.A. (2013). Selective inhibition of tumor oncogenes by disruption of super-enhancers. Cell.

[184] Yang Z., He N., Zhou Q. (2008). Brd4 recruits p-tefb to chromosomes at late mitosis to promote g1 gene expression and cell cycle progression. Mol. Cell Biol..

[185] Bandopadhayay P., Bergthold G., Nguyen B., Schubert S., Gholamin S., Tang Y., Bolin S., Schumacher S.E., Zeid R., Masoud S. (2014). Bet bromodomain inhibition of myc-amplified medulloblastoma. Clin. Cancer Res..

[186] Delmore J.E., Issa G.C., Lemieux M.E., Rahl P.B., Shi J., Jacobs H.M., Kastritis E., Gilpatrick T., Paranal R.M., Qi J. (2011). Bet bromodomain inhibition as a therapeutic strategy to target c-myc. Cell.

[187] Shao Q., Kannan A., Lin Z., Stack B.C., Suen J.Y., Gao L. (2014). Bet protein inhibitor jq1 attenuates myc-amplified mcc tumor growth in vivo. Cancer Res..

[188] Ambrosini G., Sawle A.D., Musi E., Schwartz G.K. (2015). Brd4-targeted therapy induces myc-independent cytotoxicity in gnaq/11-mutatant uveal melanoma cells. Oncotarget.

[189] Siu K.T., Ramachandran J., Yee A.J., Eda H., Santo L., Panaroni C., Mertz J.A., Sims Iii R.J., Cooper M.R., Raje N. (2016). Preclinical activity of cpi-0610, a novel small-molecule bromodomain and extra-terminal protein inhibitor in the therapy of multiple myeloma. Leukemia.

[190] Carugo A., Genovese G., Seth S., Nezi L., Rose J.L., Bossi D., Cicalese A., Shah P.K., Viale A., Pettazzoni P.F. (2016). In vivo functional platform targeting patient-derived xenografts identifies wdr5-myc association as a critical determinant of pancreatic cancer. Cell Rep..

[191] Grebien F., Vedadi M., Getlik M., Giambruno R., Grover A., Avellino R., Skucha A., Vittori S., Kuznetsova E., Smil D. (2015). Pharmacological targeting of the wdr5-mll interaction in c/ebpalpha n-terminal leukemia. Nat. Chem. Biol..

[192] Cao F., Townsend E.C., Karatas H., Xu J., Li L., Lee S., Liu L., Chen Y., Ouillette P., Zhu J. (2014). Targeting mll1 h3k4 methyltransferase activity in mixed-lineage leukemia. Mol. Cell.

